# CXCR2 inhibition enables NASH-HCC immunotherapy

**DOI:** 10.1136/gutjnl-2021-326259

**Published:** 2022-04-27

**Authors:** Jack Leslie, John B G Mackey, Thomas Jamieson, Erik Ramon-Gil, Thomas M Drake, Frédéric Fercoq, William Clark, Kathryn Gilroy, Ann Hedley, Colin Nixon, Saimir Luli, Maja Laszczewska, Roser Pinyol, Roger Esteban-Fabró, Catherine E Willoughby, Philipp K Haber, Carmen Andreu-Oller, Mohammad Rahbari, Chaofan Fan, Dominik Pfister, Shreya Raman, Niall Wilson, Miryam Müller, Amy Collins, Daniel Geh, Andrew Fuller, David McDonald, Gillian Hulme, Andrew Filby, Xabier Cortes-Lavaud, Noha-Ehssan Mohamed, Catriona A Ford, Ximena L Raffo Iraolagoitia, Amanda J McFarlane, Misti V McCain, Rachel A Ridgway, Edward W Roberts, Simon T Barry, Gerard J Graham, Mathias Heikenwälder, Helen L Reeves, Josep M Llovet, Leo M Carlin, Thomas G Bird, Owen J Sansom, Derek A Mann

**Affiliations:** 1 Newcastle Fibrosis Research Group, Biosciences Institute, Faculty of Medical Sciences, Newcastle University, Newcastle Upon Tyne, UK; 2 The Newcastle University Centre for Cancer, Newcastle University, Newcastle upon Tyne, UK; 3 Cancer Research UK Beatson Institute, Glasgow, UK; 4 MRC Centre for Inflammation Research, The Queen’s Medical Research Institute, University of Edinburgh, Edinburgh, UK; 5 Institute of Cancer Sciences, University of Glasgow, Glasgow, UK; 6 Preclinical In Vivo Imaging Facility, Faculty of Medical Sciences, Newcastle University, Newcastle upon Tyne, UK; 7 Translational Research in Hepatic Oncology, Liver Unit, IDIBAPS, Hospital Clínic, University of Barcelona, Barcelona, Spain; 8 Mount Sinai Liver Cancer Program, Division of Liver Diseases, Tisch Cancer Institute, Icahn School of Medicine at Mount Sinai, New York, New York, USA; 9 Division of Chronic Inflammation and Cancer, German Cancer Research Centre, Heidelberg, Germany; 10 Department of Pathology, Newcastle Upon Tyne Hospitals NHS Foundation Trust, Newcastle Upon Tyne, UK; 11 Flow Cytometry Facility, Biosciences Institute, Faculty of Medical Sciences, Newcastle University, Newcastle Upon Tyne, UK; 12 Innovation, Methodology and Innovation (IMA) theme, Biosciences Institute, Faculty of Medical Sciences, Newcastle University, Newcastle upon Tyne, UK; 13 Bioscience, Early Oncology, AstraZeneca, Macclesfield, UK; 14 Chemokine Research Group, Institute of Infection, Immunity and Inflammation, University of Glasgow, Glasgow, UK; 15 Department of Surgery, University Hospital Mannheim, Medical Faculty Mannheim, University of Heidelberg, Heidelberg, Germany; 16 Translational and Clinical Research Institute, Faculty of Medical Sciences, Newcastle University, Newcastle upon Tyne, UK; 17 Institució Catalana de Recerca i Estudis Avançats (ICREA), Barcelona, Spain; 18 Beatson Institute for Cancer Research, Glasgow, UK; 19 Fibrofind Ltd, William Leech Building, Medical School, Newcastle University, Newcastle upon Tyne, UK; 20 Department of Gastroenterology and Hepatology, School of Medicine, Koç University, Istanbul, Turkey

**Keywords:** hepatocellular carcinoma, immunotherapy, nonalcoholic steatohepatitis

## Abstract

**Objective:**

Hepatocellular carcinoma (HCC) is increasingly associated with non-alcoholic steatohepatitis (NASH). HCC immunotherapy offers great promise; however, recent data suggests NASH-HCC may be less sensitive to conventional immune checkpoint inhibition (ICI). We hypothesised that targeting neutrophils using a CXCR2 small molecule inhibitor may sensitise NASH-HCC to ICI therapy.

**Design:**

Neutrophil infiltration was characterised in human HCC and mouse models of HCC. Late-stage intervention with anti-PD1 and/or a CXCR2 inhibitor was performed in murine models of NASH-HCC. The tumour immune microenvironment was characterised by imaging mass cytometry, RNA-seq and flow cytometry.

**Results:**

Neutrophils expressing CXCR2, a receptor crucial to neutrophil recruitment in acute-injury, are highly represented in human NASH-HCC. In models of NASH-HCC lacking response to ICI, the combination of a CXCR2 antagonist with anti-PD1 suppressed tumour burden and extended survival. Combination therapy increased intratumoural XCR1^+^ dendritic cell activation and CD8^+^ T cell numbers which are associated with anti-tumoural immunity, this was confirmed by loss of therapeutic effect on genetic impairment of myeloid cell recruitment, neutralisation of the XCR1-ligand XCL1 or depletion of CD8^+^ T cells. Therapeutic benefit was accompanied by an unexpected increase in tumour-associated neutrophils (TANs) which switched from a protumour to anti-tumour progenitor-like neutrophil phenotype. Reprogrammed TANs were found in direct contact with CD8^+^ T cells in clusters that were enriched for the cytotoxic anti-tumoural protease granzyme B. Neutrophil reprogramming was not observed in the circulation indicative of the combination therapy selectively influencing TANs.

**Conclusion:**

CXCR2-inhibition induces reprogramming of the tumour immune microenvironment that promotes ICI in NASH-HCC.

**Significance of this study**

**What is already known on this subject?**

- Immune checkpoint inhibition (ICI) therapy is emerging as a promising new therapy for the treatment of advanced hepatocellular carcinoma (HCC).
- Only a minority of HCC patients will respond to ICI therapy and recent data suggest that HCC on the background of non-alcoholic steatohepatitis (NASH) may have reduced sensitivity to this treatment strategy.
- Neutrophils are a typical myeloid component of the liver in NASH and are found either within the HCC tumour microenvironment or in a peritumoural location.
- Neutrophils have considerable phenotypic plasticity and can exist in both tumour promoting and tumour suppressing states.
- Neutrophils may have the ability to influence ICI therapy.

**Significance of this study**

**What are the new findings?**

- CXCR2^+^ neutrophils are found in human NASH and within the tumour of both human and mouse models of NASH-HCC.
- The resistance of NASH-HCC to anti-PD1 therapy is overcome by co-treatment with a CXCR2 small molecule inhibitor, with evidence of reduced tumour burden and extended survival.
- Anti-PD1 and CXCR2 inhibitor combine to selectively reprogramme tumour-associated neutrophils (TANs) from a protumour to an anti-tumour phenotype.
- Reprogrammed TANs proliferate locally within Granzyme B^+^ immune clusters that contain physically associating CD8^+^ T cells and antigen presenting cells.
- Conventional XCR1^+^ dendritic cells are found to be elevated in anti-PD1 and CXCR2 inhibitor treated HCCs and together with CD8^+^ T cells are required for therapeutic benefit.

**How might it impact on clinical practice in the foreseeable future?**

- TANs can be selectively manipulated to adopt an anti-tumour phenotype which unlocks their potential for cancer therapy. The ability of CXCR2 antagonism to combine with ICI therapy to bring about enhanced therapeutic benefit in NASH-HCC (and potentially in HCC of other aetiologies) warrents clinical investigation.

## Introduction

Primary liver cancer is emerging globally as one of the most common and deadly malignancies with 905 000 new diagnosed cases and 830 000 deaths recorded in 2020.^1^ Hepatocellular carcinoma (HCC) accounts for up to 85% of primary liver cancers and develops on the background of chronic liver disease caused by persistent virological (hepatitis B virus (HBV) and hepatitis C virus (HCV)) or non-virological liver damage. Due to the increasing prevalence of obesity and the metabolic syndrome a high proportion of HCC is now attributed to non-alcoholic steatohepatitis (NASH), identified as the most common risk factor for HCC in UK and USA.^2 3^

Possible curative options for HCC such as tumour resection, liver transplant or ablation are at present limited to a minority of patients who are diagnosed at an early stage of the disease.^4^ For more advanced HCC, approved systemic therapies include multikinase inhibitors and agents targeting vascular endothelial growth factor (VEGF). More recently, immune checkpoint inhibition (ICI) has emerged as a therapeutic modality in HCC with PD1 antibodies (nivolumab and pembrolizumab) being approved, and a combination of anti-PDL1 (atezolizumab) with anti-VEGF (bevacizumab) now being first-line treatment for advanced HCC.^5–7^ However, only a minority (up to 30%) of HCC patients respond to immunotherapy.^5–8^ Moreover, it was recently reported that HCC on the background of NASH is less responsive to immunotherapy due to a NASH-induced alteration in the immune components of the liver and in particular an expansion in numbers of exhausted CD8^+^PD1^+^ T cells, that appear to promote, rather than suppress, NASH-HCC.^9 10^ Therefore, advanced therapeutic strategies for HCC will require a deeper appreciation of the complex immune landscape of the tumour microenvironment, and in particular, should also take into account the influence that the background liver pathology may have on the numbers, regional distributions, phenotypes and activities of key immune cell types of relevance to cancer growth.

Recent use of imaging mass cytometry (IMC) and single cell sequencing to probe the cellular constituents of human HCC revealed considerable heterogeneity within the tumour microenvironment with intratumoural region-specific distributions of immune cells.^11^ Regions with evidence of less aggressive cancer and ongoing liver damage (fibrogenesisis) were enriched for CD8^+^ T cells, B cells and CD11b^+^CD15^+^GranzymeB^+^ neutrophils. When considering the growing evidence for both pro-tumour and anti-tumour functions for neutrophils in a variety of cancers^12 13^ including HCC^14^ we were interested to determine if modulation of neutrophil biology within the tumour microenvironment would influence the resistance of NASH-HCC to anti-PD1 immunotherapy.

Here, we determined that the CXC chemokine receptor, CXCR2 is almost exclusively located on neutrophils in human and mouse NASH-HCC. This finding led us to ask if antagonism of CXCR2 can combine with anti-PD1 to overcome resistance of NASH-HCC to immunotherapy. Our findings suggest that this combination therapy reprogrammes the phenotype of tumour neutrophils and enhances their association with CD8^+^ T cells and conventional dendritic cells (cDC). Reshaping the tumour immune microenvironment was associated with a T cell- and DC-dependent reduction in tumour burden and increased survival. We propose that combined neutrophil phenotype modification and ICI may achieve improved outcomes in NASH-HCC.

### Protumour CXCR2^+^ neutrophils associate with NASH-HCC resistance to anti-PD1 immunotherapy

**Figure 1:**
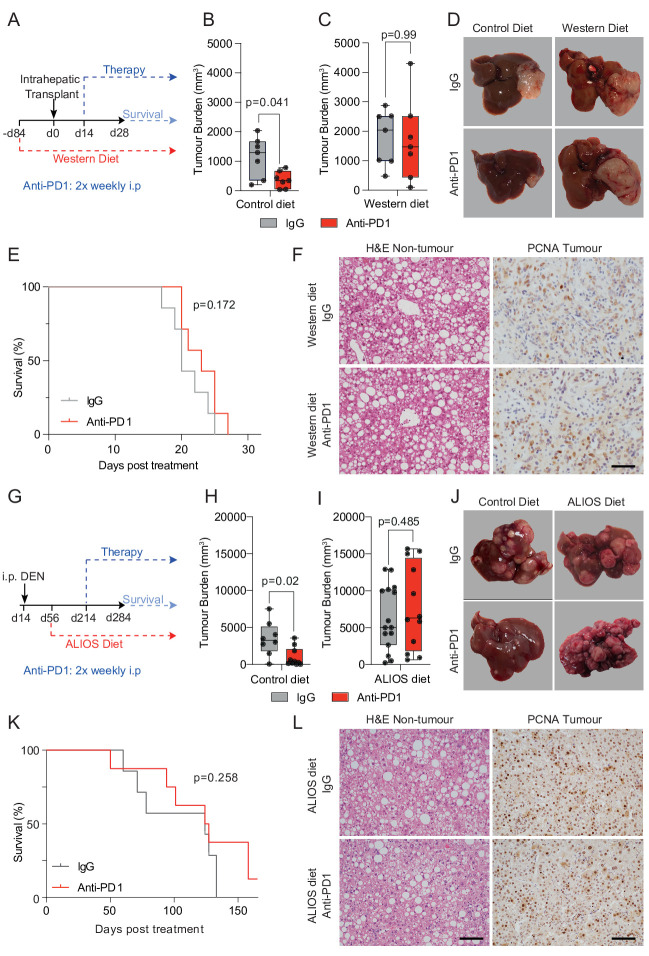
NASH-HCC is resistant to anti-PD1 immunotherapy. (A) Timeline schematic of the NASH-HCC model. (B-D) Quantification and representative images of tumour burden at day 28 post-intrahepatic injection for orthotopic HCC mice fed a control diet or western diet and treated with IgG-control or anti-PD1. (E) Survival plot in orthotopic NASH-HCC mice fed a Western diet and treated with IgG-control or anti-PD1. (F) Representative images of H&E-stained non-tumour livers and PCNA-stained tumours from orthotopic NASH-HCC mice fed a Western diet and treated with IgG-control or anti-PD1. Scale bar = 100 µm. (G) Timeline schematic for the DEN/ALIOS NASH-HCC model. (H-J) Quantification and representative images of tumour burden at day 284 for DEN mice fed a control diet or ALStreated with IgG-control or anti-PD1. (K) Survival plot in DEN/ALIOS mice treated with IgG-control or anti-PD1 (censored at day 165 post-treatment). (L) Representative images of H&E-stained non-tumour livers and PCNA-stained tumours from DEN/ALIOS mice treated with IgG-control or anti-PD1 at day 284. Scale bar = 100 µm. Dots in (B, C, H, I) represent individual mice. Significance tested using: Mann-Whitney*U*-test (A, B, H, L) and Log-rank (Mantel-Cox) test (E, K). Exact p-values indicated on graph. HCC, hepatocellular carcinoma; NASH, non-alcoholic steatohepatitis; PCNA, proliferating cell nuclear antigen.

To investigate the immunological determinants of unresponsiveness of NASH-HCC to anti-PD1 therapy we designed an orthotopic mouse model using the Hep-53.4 HCC cell line, which was selected due to its high mutational burden (online supplemental figure 1A–C). On the background of steatosis induced by a modified diet of high sugar and fat, we observed weight gain and larger tumours compared with non-steatotic controls (online supplemental figure 1D, E). Tumours in non-steatotic controls were responsive to anti-PD1 therapy, however, anti-PD1 showed no benefit on tumour burden, survival, steatosis, proliferation or immune cell infiltration in steatotic mice (figure 1A–F and online supplemental figure 1H, K). For an additional autochthonous model, we employed either Diethylnitrosamine (DEN) alone or in combination with the American lifestyle induced obesity syndrome diet (DEN/ALIOS), the latter to establish HCC on a background of NASH^15 16^ (online supplemental figure 1I–O). Anti-PD1 responsiveness was observed in DEN mice fed a control diet, whereas anti-PD1 therapy had no effect on tumour burden, proliferation, or steatosis and animal survival when mice were fed the ALIOS diet (figure 1G–L and online supplemental figure 1P, Q). However, F4/80^+^ and CD3^+^ immune cell infiltrates were increased in anti-PD1 treated ALIOS fed mice (online supplemental figure 1R, S) indicative of anticipated alterations in tumour immunity.

10.1136/gutjnl-2021-326259.supp1 Supplementary data

**Figure 2:**
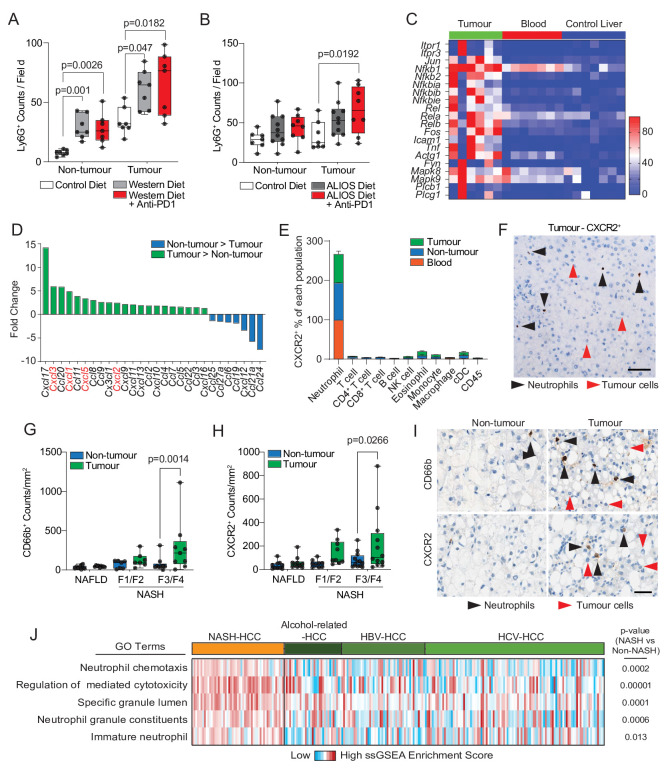
NASH-HCC and anti-PD1 resistance is associated with CXCR2^+^ neutrophils. (A) Quantification of Ly6G^+^ counts/field in non-tumour liver and tumours from IgG-control or anti-PD1 treated orthotopic NASH-HCC mice. (B) Quantification of Ly6G^+^ counts/field in non-tumour liver and tumours from IgG-control or anti-PD1 treated DEN/ALIOS mice. (C) Heatmap showing row-scaled expression of DEGs associated with a pro-tumour neutrophil phenotype upregulated in DEN/ALIOS TANs compared with peripheral blood and control liver neutrophils. (D) Quantification of fold change for *Cxcl* and *Ccl* chemokine transcripts between DEN/ALIOS non-tumour liver and tumour. (E) Flow cytometric quantification of CXCR2^+^ as a percentage of cell populations in the peripheral blood, non-tumour liver and tumour in DEN/ALIOS mice. Error bars represent Mean ± SEM. (F) Representative image of RNAscope *in situ* hydrisation staining of CXCR2 in DEN/ALIOS mouse tumours. Black arrowheads indicate positive infiltrating non-parenchymal cells and red arrows indicate negative tumour cells. (G-I) Quantification and representative images of CD66b^+^ and CXCR2^+^ cell counts/mm^2^ in non-tumour liver and tumour by IHC of non-alcoholic fatty liver disease (NAFLD)-HCC and NASH-HCC patient resected tissue. (J) Heatmap showing row-scaled expression of neutrophil-associated process networks for human NASH-HCC compared with HBV, HCV and alcohol-related HCC (non-NASH-HCC). Scale bar = 100 µm. Data are from; bulk Ly6G^+^ neutrophil RNA-Seq (C), bulk tissue RNA-Seq (D), bulk tumour microarray (J). Dots in (A, B, G, H) represent individual mice. Significance tested using: Two-way ANOVA with Sidak’s multiple comparisons test (A, B, G, H). Exact p-values indicated on graph. ALIOS, American lifestyle induced obesity syndrome diet; ANOVA, analysis of variance; DEN, Diethylnitrosamine; GO, gene ontology; HBV, hepatitis B virus; HCC, hepatocellular carcinoma; HCV, hepatitis C virus; IHC, immunohistochemistry; NASH, non-alcoholic steatohepatitis; TANs, tumour-associated neutrophils.

Although elevated numbers of circulating neutrophils are associated with reduced HCC survival,^17^ by contrast an enrichment of tumour-associated neutrophils (TANs) is reported to correlate with improved survival.^18^ This latter observation indicates a potential for TANs to influence the progression of HCC and raises the question of whether immunotherapy is influencing TANs (and vice versa). Ly6G^+^ neutrophils were found to be present in both tumour and non-tumour tissue of orthotopic-HCC mice and were significantly elevated in both compartments in the presence of NASH and remained high with anti-PD1 therapy (figure 2A and online supplemental figure 2A). Increased numbers of TANs were also a feature in the DEN/ALIOS model and the increase reached significance with anti-PD1 treatment (figure 2B and online supplemental figure 2A). In addition, TANs were elevated in choline deficient-high fat diet (CD-HFD) spontaneous NASH-HCC model, and were retained with anti-PD1 therapy which is reported to also fail in this model^9^ (online supplemental figure 2B, C). Thus, we consistently observe TANs to accumulate in NASH-HCC, independent of the model examined, and they are retained in the tumour with anti-PD1 therapy. TANs display functional heterogeneity including anti-tumour or protumour phenotypes that impact on tumour growth.^19^ Using transcriptomic profiling of tumour-isolated Ly6G^+^ cells, we determined the phenotype of TANs from DEN/ALIOS tumours. To account for environmentally induced differences in gene expression,^20^ we compared TANs with peripheral blood and liver neutrophils. DEGs with increased expression were enriched for process networks associated with inflammatory (eg, *Nfkb1/Rel*, *Mapk8/Jnk1*, *Mapk9/Jnk2*, *Icam1*) and calcium (eg, *Itpr1, Plcb1, Plcg1*) signalling (figure 2C and online supplemental figure 2D). Genes associated with a protumour neutrophil phenotype, including *Csf1, Ccl3, Vegfa* and *Ptgs2* 19–21 were also significantly upregulated in TANs (online supplemental figure 2E).

Transcriptomic analysis of DEN/ALIOS tumours identified an upregulation of myeloid associated cytokine and chemokine gene expression compared with normal liver (figure 2D). Notably, ligands (*Cxcl1, Cxcl2, Cxcl3, Cxcl5*) for the chemokine receptor CXCR2, the latter identified as being predominantly expressed by Ly6G^+^ neutrophils, were all increased in tumour tissue (figure 2D, E and online supplemental figure 2F). In situ hybridation analysis of *Cxcr2* expression in DEN/ALIOS mouse tumours confirmed expression of *Cxcr2* to be specifically associated with morphologically identified infiltrating neutrophils and absent in parenchymal and tumour cells (figure 2F). This identifies CXCR2 as a neutrophil chemokine receptor that could be targeted to manipulate TANs in models of HCC-NASH.^14^ In humans, the CXCR2 ligands *CXCL1* and *CXCL8* were significantly upregulated in NASH-HCC compared with NASH (online supplemental figure 2G). Neutrophil chemotaxis/migratory gene ontology terms were enriched in advanced human NASH (F4 fibrosis)^22^ and numbers of hepatic CD66b^+^ neutrophils increased with severity of NASH (online supplemental figure 2H, I). Moreover, in HCC patient tissue, CD66b^+^ neutrophils and CXCR2^+^ cells predominantly localised to NASH-HCC tumours with expression of the two markers correlating, and furthermore being demonstrated to be colocalised at the cellular level (figure 2G, H and online supplemental figure 2J, K). Similar to the mouse models, CXCR2 expression was limited to infiltrating immune cells and was absent on tumour epithelium within HCC in patients (figure 2I). We additionally noted that neutrophil expression signatures were enriched in human NASH-HCC compared with HBV-HCC, HCV-HCC and alcohol-related-HCC^23^ (figure 2J). Thus, tumour infiltration of CXCR2-expressing neutrophils is characteristic of both murine models and human NASH-HCC and associates with resistance to anti-PD1 therapy in experimental models of NASH-HCC.^9^

### CXCR2 antagonism resensitises NASH-HCC to immunotherapy

We next determined the effects of a CXCR2 small molecule inhibitor (AZD5069)^24^ in experimental NASH-HCC either administered alone or in combination with anti-PD1. We hypothesised that AZD5069 would suppress hepatic neutrophil recruitment. This was confirmed in the context of DEN-induced acute liver damage (online supplemental figure 3A–C). We also observed no change in F4/80^+^ macrophages and CD3^+^ T cells (online supplemental figure 3D, E). These data are consistent with previous studies showing that in acute inflammatory settings CXCR2 inhibition selectively reduces neutrophil recruitment.^24^

**Figure 3:**
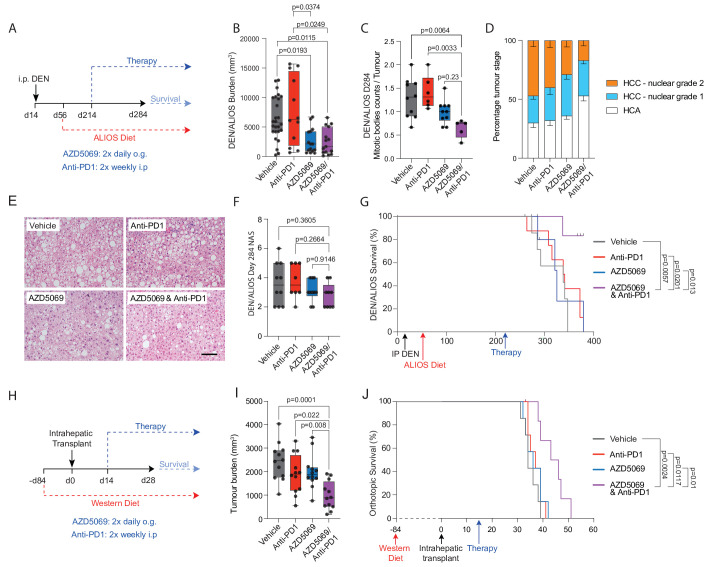
Inhibition of CXCR2^+^ protumour neutrophils resensitises NASH-HCC to anti-PD1 therapy. (A) Schematic for DEN/ALIOS model treatment regime. (B) Quantification of tumour burden for DEN/ALIOS mice at day 284 for each treatment arm. (C) Quantification of average mitotic body counts per tumour for DEN/ALIOS mice at day 284. (D) Quantification of tumour stage based on nuclear grading for DEN/ALIOS mice at day 284 for each treatment arm. Mean ± SEM. (E) Representative images of non-tumour liver H&E for DEN/ALIOS mice. Scale bar = 100 µm. (F) Quantification of NAFLD activity score (NAS) in the livers for DEN/ALIOS mice at day 284. G) Survival plot for DEN/ALIOS mice (censored at day 365). (H) Schematic for orthotopic NASH-HCC model treatment regime. (I) Quantification of tumour burden for the orthotopic NASH-HCC mice at day 28. (J) Survival plot in orthotopic NASH-HCC mice. One mouse censored due to non-liver related medical issue. Dots in (B, C, F, I) represent individual mice.Significance tested using: Kruskal-Wallis test with Dunn’s multiple comparisons test (B), One-Way ANOVA with Tukey multiple comparisons test (C, F, I), Log-rank (Mantel-Cox) test (G, J). Exact p-values indicated on graph. ALIOS, American lifestyle induced obesity syndrome diet; ANOVA, analysis of variance; DEN, Diethylnitrosamine; HCC, hepatocellular carcinoma; NAFLD, non-alcoholic fatty liver disease; NASH, non-alcoholic steatohepatitis.

Treatments using either or both AZD5069/anti-PD1 were then investigated for their ability to suppress tumour growth in the DEN-ALIOS model (figure 3A). Tumour burden at day 284 was reduced for AZD5069 monotherapy and with combined AZD5069/anti-PD1 treatment compared with vehicle and anti-PD1 monotherapy, however, no change in tumour number was identified suggesting a suppression of cancer progression (figure 3B and online supplemental figure 3F, G). Examination of tumours revealed reduced numbers of epithelial mitotic bodies and a lower tumour-stage grading for the AZD5069/anti-PD1 group compared with other treatment arms including AZD5069 monotherapy without significantly altering the underlying NASH pathology (figure 3C–F and online supplemental figure 3H). This is clinically relevant as a high mitotic index in human HCC is a predictor of shorter disease-specific survival.^25^ It was therefore noteworthy that the combination of AZD5069/anti-PD1 improved survival relative to monotherapies (figure 3G) Importantly, the benefits of AZD5069/anti-PD1 therapy were recapitulated in the orthotopic NASH-HCC model (figure 3H–J). In contrast to the DEN/ALIOS model, a lack of therapeutic effect was observed with either AZD5069 or anti-PD1 monotherapy (figure 3I, J). However, AZD5069/anti-PD1 combination therapy reduced tumour burden at day 28 and extended survival relative to vehicle control and monotherapies (figure 3I, J and online supplemental figure 3I). Notably, the treatments had no influence on steatosis or body weight (online supplemental figure 3J, K). AZD5069/anti-PD1 treated mice reached clinical endpoint later, at which point tumour burden was similar between treatment arms, this being consistent with suppression of tumour growth (online supplemental figure 3L). Hence, although CXCR2 antagonism alone delivered modest model-dependant antitumour benefit, similar to observations made in models of non-hepatic cancers,^26–31^ we show that CXCR2 inhibition sensitises to anti-PD1 immunotherapy in models of NASH-HCC that are otherwise resistant to anti-PD1 monotherapy.

### AZD5069/anti-PD1 therapy promotes an antitumour immune microenvironment

**Figure 4:**
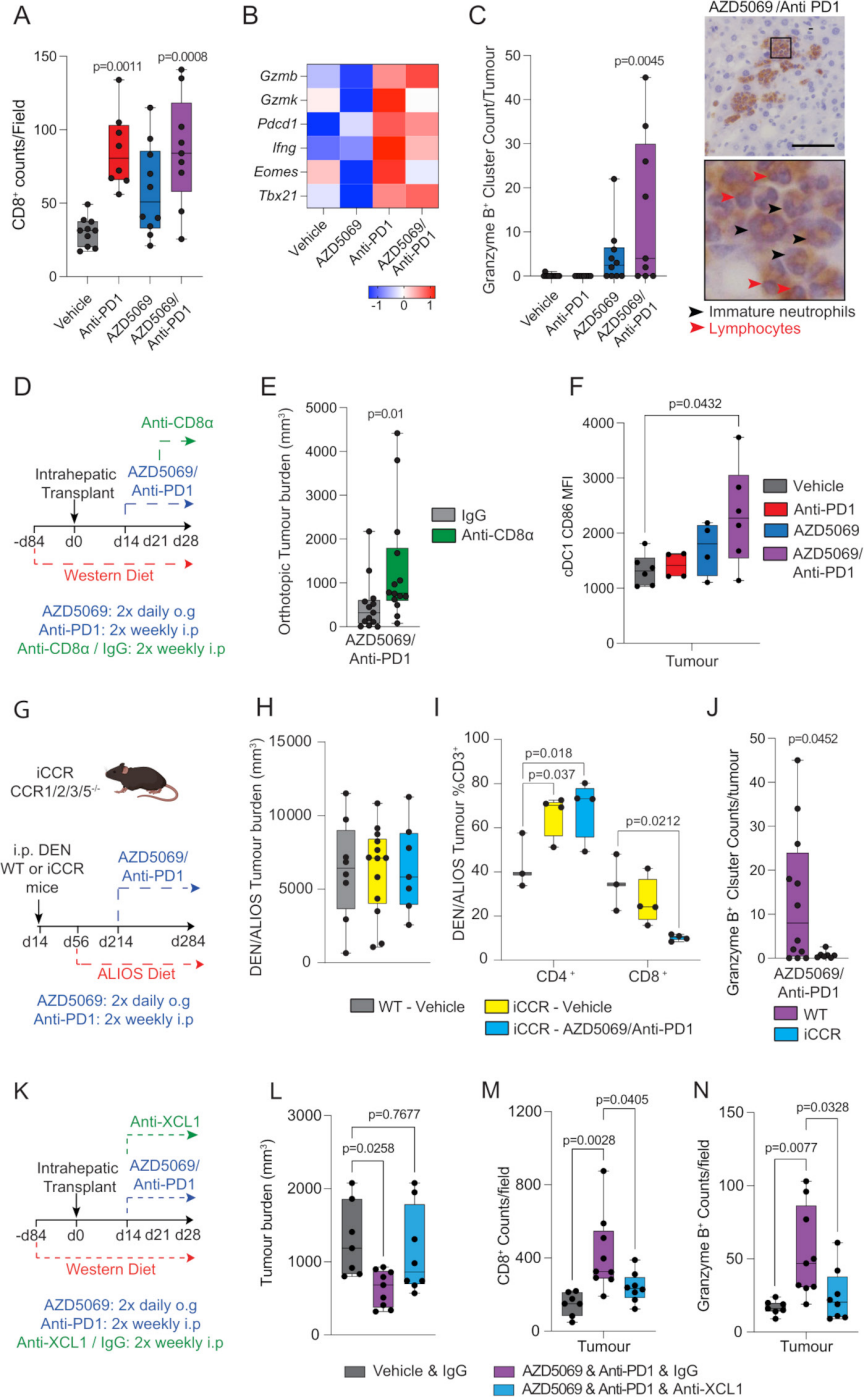
AZD5069/anti-PD1 therapy promotes an anti-tumour immune microenvironment. (A) Quantification of CD8^+^ counts/field in tumours DEN/ALIOS model from each treatment arm. (B) Heatmap showing row-scaled expression of genes associated with CD8^+^ T cell activation and exhaustion for DEN/ALIOS treatment groups. Data are from bulk CD3^+^ Tumour associated T cells analysed by RNA-Seq. (C) Quantification of granzyme B^+^clusters in the tumours for DEN/ALIOS mice from each treatment arm at day 284 and representative images of granzyme B^+^ clusters in AZD5069/anti-PD1 treated mice (black arrow heads = banded neutrophils; blue arrow heads = lymphocytes). Scale bar = 100 µm. (D) Timeline schematic for the anti-CD8a depletion regime in the orthotopic NASH-HCC model. (E) Quantification of tumour burden in orthotopic NASH-HCC mice treated with AZD5069/anti-PD1 and IgG-control or anti-CD8α at day 28 post-intrahepatic injection. (F) Flow cytometric quantification of CD86 median fluorescence intensity (MFI) of intratumoural XCR1^+^ cDC1 cells from DEN/ALIOS mice treatment arms at day 284. (G) Timeline schematic for the DEN/ALIOS regimen and targeted therapies in mice with compound deletion of *Ccr1, 2, 3, 5* knockout mice, designated iCCR. (H) Quantification of tumour burden for DEN/ALIOS mice Vehicle-treated WT and iCCR, and AZD5069/anti-PD1 treated iCCR mice at day 284. (I) Flow cytometric quantification of CD4^+^ and CD8^+^ cells as a percentage of CD3^+^ T cells in tumours from WT-Vehicle, iCCR-Vehicle and iCCR-AZD5069/anti-PD1 treated DEN/ALIOS mice at day 284. (J) Quantification of granzyme B^+^ clusters in WT and iCCR DEN/ALIOS mice treated with AZD5069/Anti-PD1 at day 284. (K) Timeline schematic for the anti-XCL1 neutralisation regime in the orthotopic NASH-HCC model. (L) Quantification of tumour burden in orthotopic NASH-HCC mice treated with vehicle control and IgG-control or AZD5069/anti-PD1 and either IgG-control or anti-XCL1 at day 28 post-intrahepatic injection. (M, N) Quantification of CD8^+^ and granzyme B^+^ counts/field in tumours of orthotopic NASH-HCC mice treated with vehicle control and IgG-control or AZD5069/anti-PD1 and either IgG-control or anti-XCL1 at day 28 post-intrahepatic injection. Dots in (A, C, E, F, H-J, L-N) represent individual mice. Significance tested using: One-Way ANOVA with Tukey multiple comparisons test (A, C, F, L, M, N), Mann-Whitney *U*-test (E), Two-way ANOVA with Tukey’s multiple comparisons test (I), Unpaired T-test (J). Exact p-values indicated on graph. ALIOS, American lifestyle induced obesity syndrome diet; ANOVA, analysis of variance; DEN, Diethylnitrosamine; HCC, hepatocellular carcinoma; IHC, immunohistochemistry, NASH, non-alcoholic steatohepatitis; WT, wild-type.

To further examine the concept that CXCR2 antagonism sensitises NASH-HCC to anti-PD1 therapy we asked if combination therapy activates classic T-cell mediated anti-tumour immunity. Characterisation of intratumoural T cells revealed intratumoural CD8^+^ T cells were significantly increased in both anti-PD1 and AZD5069/anti-PD1 therapy groups, with only anti-PD1 monotherapy significantly affecting CD4^+^ T cells (figure 4A and online supplemental figure 4A). Combination therapy also enhanced intratumoural CD8^+^ T cell numbers in the orthotopic model (online supplemental figure 4B) Flow cytometric analysis revealed no gross phenotypic changes in early effector CD8^+^CD44^Hi^ T cells across treatment groups. However, anti-PD1 treatment alone significantly increased numbers of CD8^+^PD1^+^ T cells, this effect being recently reported by Pfister *et al* 9 who suggested this T cell phenotype compromises the efficacy of anti-PD1 treatment in NASH-HCC (online supplemental figure 4C). The percentage of CD4^+^PD1^+^ T cells was also higher in the context of anti-PD1 monotherapy relative to other treatment groups (online supplemental figure 4D). RNAseq on isolated CD3^+^ cells revealed enhanced expression of the recently identified T cell ageing markers *Gzmk* and *Eomes* following anti-PD1 therapy, both of which were suppressed when AZD5069 was combined with anti-PD1 (figure 4B). Alongside these changes, AZD5069/anti-PD1 therapy enhanced the expression of Granzyme B (Gzmb), a cytotoxic serine protease expressed by neutrophils, NK cells and by recently activated CD8^+^ T cells and for which expression correlates with clinical outcome in PD1 immunotherapy.^32–35^ Immunostaining of DEN/ALIOS tumours revealed that Gzmb was detected at low levels in vehicle and monotherapy groups yet in the context of AZD5069/anti-PD1 combination therapy was highly expressed and was localised within discrete immune cell clusters containing banded immature neutrophils and lymphocytes (figure 4C and online supplemental figure 4E). Enhanced Gzmb protein expression was also achieved with combination therapy in orthotopic tumours where we also noted that anti-PD1 monotherapy depressed expression of the protease relative to vehicle control (online supplemental figure 4F). These data led us to ask if depletion of CD8^+^ T cells would modulate the anti-tumour effects of AZD5069/anti-PD1 therapy. Depletion of CD8^+^ T cells was carried out by administration of anti-CD8α to mice bearing an orthotopic NASH-HCC tumour and alongside AZD5069/anti-PD1 treatment (figure 4D). Succesful depletion of CD8^+^ T cells was confirmed by an increase in the proportion of CD4^+^ cells relative to the total CD3^+^ population (online supplemental figure 4G–I) and resulted in a higher orthotopic tumour burden compared with IgG controls (figure 4E). The requirement for CD8^+^ T cells for the anti-tumour effect of combination therapy was additionally confirmed by performing anti-CD8α-mediated depletion in tumour-bearing DEN/ALIOS mice treated with combination AZD5069/anti-PD1 (online supplemental figure 4J–M).

As recruitment and activation of XCR1^+^ cDC1 in tumours is considered critical for activation of cytotoxic CD8^+^ T cells and immunotherapy^36^ we next assessed CD86 surface expression as a marker of cDC1 activation in mice treated with AZD5069/anti-PD1 therapy. Anti-PD1 alone had no effect on activation of intratumour XCR1^+^ cDC1 cells compared with vehicle controls in the DEN/ALIOS model (figure 4F). AZD5069 alone also had no effect on activation of intratumour XCR1^+^ cDC1 cells, likely due to the limited expression of CXCR2 on cDCs (figure 2E). However, combined AZD5069/anti-PD1 therapy substantially increased the activation of intratumoural cDC1 cells (figure 4F). As several CC chemokines associated with DC recruitment were expressed in mouse NASH-HCC tumours responding to mono and dual therapies (online supplemental figure 5A) we next determine the effects of perturbing DC recruitment employing mice that are deficient for *Ccr1, Ccr2, Ccr3* and *Ccr5*, termed iCCR.^37^ These mice were treated as per the DEN/ALIOS model and AZD5069/anti-PD1 or control therapy administered (figure 4G). The number of cDC1 and cDC2 cells, and to a lesser extent F4/80^+^ macrophages but not neutrophils, were decreased in the tumours of iCCR mice (online supplemental figure 5B–D). Importantly, loss of myeloid recruitment alone in iCCR mice had no impact on tumour burden in the DEN/ALIOS model (figure 4H). However, unlike in wild-type mice, AZD5069/anti-PD1 therapy failed to reduce tumour burden in iCCR mice (figure 4H). This loss of effect was associated both with a reduction in tumour associated CD3^+^CD8^+^ T cells and loss of granzyme B^+^ immune clusters (figure 4I.J). To corroborate these data and to more specifically address the role of XCR1^+^ cDC1 cells we determined if AZD5069/anti-PD1 therapy of orthotopic NASH-HCC would be affected by anti-XCL1-mediated blockade of XCL1, a major chemokine involved in mediating cDC1 and CD8 T cell interactions (figure 4K).^38^ AZD5069/anti-PD1 therapy resulted in an increase in activated intratumoural XCR1^+^ cDC1 cells in line with observations in DEN/ALIOS mice, but with cDC1 activation being selectively suppressed on treatment with anti-XCL1 (online supplemental figure 5E, F). This effect was associated with loss of the anti-tumoural action of AZD5069/anti-PD1 therapy; demonstrated by increased tumour burden in anti-XCL1 treated mice compared with IgG controls (figure 4L). Confirming an associated impact on cytotoxic T cells, AZD5069/anti-PD1-induced increases in intratumoural cytotoxic CD8^+^ and GzmB^+^ cells which was suppressed when cDC1 activation was selectively blocked by anti-XCL1 (figure 4M, N). We conclude that combined suppression of CXCR2 and PD1 stimulates both intratumoural recruitment and activation of cDC1 cells enabling T cell-mediated cytotoxicity.

### AZD5069/anti-PD1 therapy promotes tumour neutrophil accumulation and the formation of intratumoural immunological hubs

**Figure 5:**
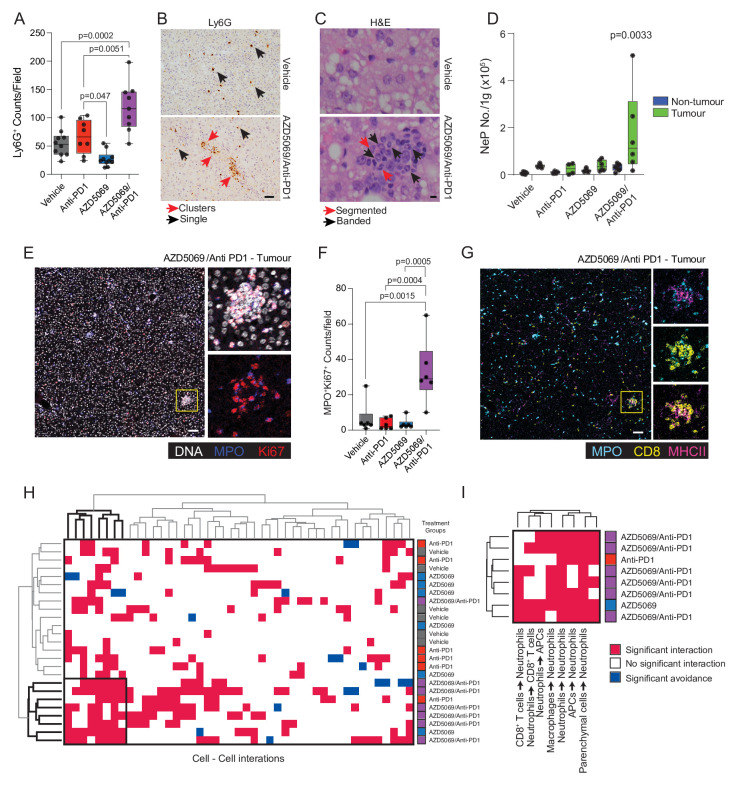
AZD5069/anti-PD1 therapy promotes tumour neutrophil accumulation and the formation of intratumoural immunological hubs. (A) Quantification of Ly6G^+^ counts/field by IHC for DEN/ALIOS mice tumours at day 284. (B) Representative images of Ly6G^+^ staining for DEN/ALIOS tumours in mice treated with vehicle or AZD5069/Anti-PD1. Black arrows indicate single Ly6G^+^ neutrophils; red arrows indicate clusters of Ly6G^+^ neutrophils. Scale bar = 100 µm. (C) Representative H&E from Vehicle-control and AZD5069/anti-PD1 treated mouse tumours identifying clusters of neutrophils with banded (blue arrows) and segmented (black arrows) nuclear morphology. Scale bar = 10 µm. (D) Flow cytometric quantification of NeP count/gram in non-tumour liver and tumour tissues for DEN/ALIOS mice for each treatment arm at day 284. (E) Representative intra-tumour IMC image for DEN/ALIOS mice treated with AZD5069/anti-PD1. DNA = white; MPO = blue; Ki-67 = red. n=6 mice. Scale bar = 100 µm. (F) Quantification of MPO^+^Ki-67^+^ counts/field for DEN/ALIOS tumours from IMC analysis. (G) Representative intra-tumour IMC image for DEN/ALIOS mice treated with AZD5069/anti-PD1. MPO = cyan; CD3 = yellow; MHCII = purple. Scale bar = 100 µm. (H) HistoCAT neighbourhood clustering analysis performed using phonograph clustered cell populations across all four treatment arms where red indicated a significant interaction, blue indicates a significant avoidance and white indicated no significant interaction. Each column represents the interaction of two cell types. Each row represents an individual mouse. (I) Magnified image of HistoCAT neighbourhood clustering analysis. Cluster showing specifically enriched cell-cell interactions. 7/8 cell-cell interactions characterised by antibodies used. Dots in (A, D, F) represent individual mice. Significance tested using: One-Way ANOVA with Tukey’s multiple comparisons test (A, F), Two-way ANOVA with Sidak’s multiple comparisons test (D). Exact p-values indicated on graph. ALIOS, American lifestyle induced obesity syndrome diet; ANOVA, analysis of variance; APC, antigen presenting cell; DEN, Diethylnitrosamine; HCC, hepatocellular carcinoma; IHC, immunohistochemistry, IMC, imaging mass cytometry; NASH, non-alcoholic steatohepatitis.

Given that CXCR2 is almost exclusively expressed on neutrophils (figure 2E), we were curious as to their role in AZD5069/anti-PD1 therapy and its associated tumour immune cell remodelling. Unexpectedly, we observed that combination therapy in both models of NASH-HCC was associated with a dramatic increase in TANs, whereas AZD5069 monotherapy brought about the anticipated reduction in TANs (figure 5A, B and online supplemental figure 6A). Real-time analysis of tumour neutrophil infiltration was not possible across the therapy time-course, so instead we examined circulating neutrophils sampled weekly from peripheral blood. Anti-PD1 alone had no demonstrable effects on circulating neutrophil numbers across the treatment period, whereas AZD5069 stimulated a transient increase in circulating Ly6G^+^ neutrophils peaking at 4 weeks after start of treatment (online supplemental figure 6B). A similar transient increase in circulating neutrophils was observed in AZD5069/anti-PD1 treated mice, however, this effect was delayed peaking at 6 weeks from start of treatment. These peripheral blood data indicated a change in neutrophil behaviour in response to dual long-term targeting of CXCR2 and PD1.

Immunohistochemical analysis of tumours identified clusters of TANs that were unique to AZD5069/anti-PD1 treatment and comprising a mixed population of banded and segmented neutrophil populations (figure 5B, C and online supplemental figure 6C). The presence of these clustered TANs in AZD5069/anti-PD1 treated HCCs was intriguing and suggestive of local proliferation. Zhu *et al* 39 recently described early unipotent neutrophil progenitors (NeP) that produce neutrophils from adult bone marrow (BM). Conspicuously, NePs were significantly increased not only in the BM but also in tumours of AZD5069/anti-PD1 treated mice (figure 5D and online supplemental figure 6D, E). AZD5069/anti-PD1 treatment, therefore, alters granulopoiesis, while intratumour NePs may locally generate neutrophils, thus offering an explanation for the unexpectedly elevated numbers of TANs observed in mice receiving combination therapy.

To validate the presence of immature neutrophils in combined AZD5069/anti-PD1 treated tumours we used IMC of tumour sections from DEN/ALIOS treatment arms (online supplemental figure 6F–J). Neutrophils, both immature and mature, were identified as expressing the primary granule protein MPO. We confirmed intratumoural clusters of proliferating MPO^+^Ki67^+^ neutrophils to be significantly increased in AZD5069/anti-PD1 treated mice compared with monotherapies and vehicle controls (figure 5E, F). IMC neighbourhood analysis revealed intimate associations of MPO^+^Ki67^+^ neutrophils with CD8^+^ T cells and MHC Class II^+^ (MHCII^+^) antigen presenting cells (APCs) that were found in the regions of interest with six out of seven AZD5069/anti-PD1 treated tumours that were examined by IMC (figure 5G–I). In contrast, for anti-PD1 and AZD5069 monotherapies, IMC only detected these mixed immune cell hubs in one tumour for each type of treatment (figure 5I).

Intravital microscopy confirmed the presence of stable tumour-associated Ly6G^+^ clusters, in vivo, in AZD5069/anti-PD1 treated mice (online supplemental figure 6K, L). Directly interacting Ly6G^+^ TANs and CD3^+^CD8^+^ T cells that maintained physical contact over several minutes or more were also observed (online supplemental figure 6M).

Longitudinal imaging of ex vivo precision cut liver slices (PCLS) was then performed to further interrogate Ly6G^+^ cell (neutrophil), CD8^+^ cell (T cell) and CD11c^+^ cell (DC and a subset of macrophages) dynamics within the tumours of DEN/ALIOS mice (online supplemental figure 6N, O and video). PCLS from AZD5069/anti-PD1 treated mice had the expected, elevated numbers of neutrophils, CD11c^+^ cells and CD8^+^ T cells (online supplemental figure 6P–R). Although T cell speeds remained low in PCLS from all groups, neutrophil speed was increased in AZD5069/anti-PD1 treated tumours suggesting a more actively migrating phenotype for these neutrophils (online supplemental figure 6S, T). Neutrophil-CD11c^+^ cell interactions were high in tumours irrespective of treatment, however, neutrophil-CD8^+^ T cell and CD11c^+^-CD8^+^ T cell interactions were elevated in AZD5069/anti-PD1 treated tumours compared with vehicle controls (online supplemental figure 6U–Y, video). These data provide evidence that combined therapeutic targeting of CXCR2^+^ neutrophils and the PD1-PDL1 immune checkpoint remodels the NASH-HCC tumour immune microenvironment, including the generation of locally proliferating immature NeP in close physical association with cytotoxic T cells.

10.1136/gutjnl-2021-326259.supp4 Supplementary video

### AZD5069/anti-PD1 combination therapy reprogrammes the TAN phenotype

**Figure 6:**
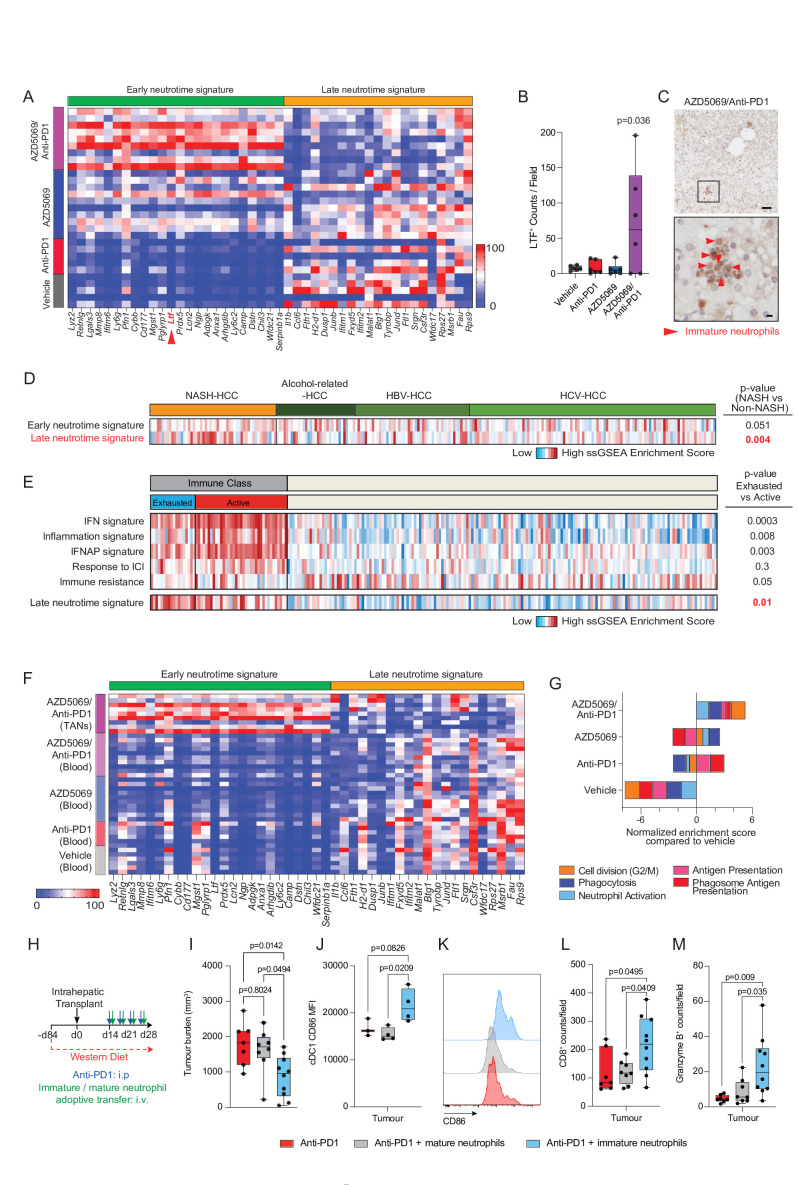
AZD5069/anti-PD1 combination therapy reprogrammes the TAN phenotype. (A) Heatmap showing row-scaled expression of genes associated with late and early neutrotime for DEN/ALIOS mice TANs. (B) Quantification of LTF^+^ counts/field by IHC for DEN/ALIOS mice tumours at­ day 284. (C) Represenative image of LTF positive neutrophils (red arrow) in the tumour of AZD5069/anti-PD1 treated DEN/ALIOS mice at day 284. Scale bar top = 100 µm, bottom = 10 µm. (D) Heatmap showing row-scaled expression of early and late neutrotime signatures for human NASH-HCC compared with HBV, HCV and alcohol-related HCC (non-NASH-HCC). In total n=237 patients analysed. (E) Heatmap showing row-scaled expression of published HCC immune class signatures; IFN, inflammation, IFNAP, Response to ICI and immune resistance as well as the late neutrotime signature for human HCC active and exhausted immune subsets. In total n=228 patients analysed. (F) Heatmap showing row-scaled expression of genes associated with late and early neutrotime signatures for DEN/ALIOS peripheral blood neutrophils and AZD5069/Anti-PD1 treated TANs. (G) Gene set enrichment analysis (GSEA) showing normalised enrichment scores for TAN process networks highly enriched in; Anti-PD1 vs Vehicle (Phagosome Antigen Presentation and Antigen Presentation), AZD5069 vs Vehicle (Neutrophil Activation and Phagocytosis), and AZD5069/Anti-PD1 vs Vehicle (G2-M). (H) Timeline schematic for neutrophil based therapy treatment regime in the orthotopic NASH-HCC model. (I) Quantification of tumour burden in orthotopic NASH-HCC mice treated with anti-PD1 and immature or mature neutrophils at day 28 post-intrahepatic injection. (J, K) Flow cytometric quantification and representative histogram plot of CD86 median fluorscent intensity (MFI) of intraturmoural XCR1^+^ cDC1 cells from orthotopic NASH-HCC neutrophil/anti-PD1 therapy mice at day 28. (L, M) Quantification of intratumoural CD8^+^ and gramzyme B^+^ counts/field in the tumours of orthotopic NASH-HCC neutrophil/anti-PD1 therapy mice at day 28. Data are from: Bulk DEN/ALIOS Ly6G^+^ TAN RNA-Seq data in (A, F, G) and bulk tumour microarray in (D, E). Dots in (B, I, J, L, M) represent individual mice. Significance tested using: One-Way ANOVA with Tukey’s multiple comparisons test (B, I, J, L, M). Exact p-values indicated on graph. ALIOS, American lifestyle induced obesity syndrome diet; ANOVA, analysis of variance; DEN, Diethylnitrosamine; HCC, hepatocellular carcinoma; IHC, immunohistochemistry; ICI, immune checkpoint inhibition; NASH, non-alcoholic steatohepatitis; TANs, tumour-associated neutrophils.

Given that our observations were consistent with intratumoural granulopoiesis in response to combination AZD5069/anti-PD1 therapy, we more closely characterised the TAN phenotype under these conditions. Grieshaber-Bouyer *et al* 40 recently reported a chronologically ordered developmental path for neutrophils termed ‘neutrotime’. This extends from immature preneutrophils (early neutrotime) that are predominantly found in BM to fully mature neutrophils (late neutrotime) mainly located in the circulation and spleen (online supplemental figure 7A). TAN transcriptome analysis revealed that AZD5069/anti-PD1 therapy induced neutrotime reprogramming along this neutrotime spectrum (figure 6A and online supplemental figure 7B). TANs in vehicle, anti-PD1 and AZD5069 treated tumours phenotypically resembled mature neutrophils, expressing genes characteristic of the late neutrotime (eg, *Jund, Csf3r*, *Rps27*) (figure 6A and online supplemental figure 7B). However, late neutrotime genes were comprehensively downregulated in TANs from AZD5069/anti-PD1 treated mice, with a corresponding upregulation of transcripts characteristic of the early neutrotime (eg, *Mmp8, Retnlg, Ltf, Lcn2, Camp, Chil3, Tuba1b*, *Fcnb*). Lactoferrin (Ltf) was of particular interest among the early neutrotime genes as its protein has well documented anti-cancer activities; including the activation of DCs and macrophages and enhancing the cytotoxic properties of natural killer cells.^41–43^ Staining for Lactoferrin in DEN/ALIOS tumours was elevated in AZD5069/anti-PD1 treated mice where the protein was localised to the neutrophil-rich immune clusters that included banded immature neutrophils (figure 6B, C). These observations indicate a potential mechanism by which reprogrammed TANs may network with other immune cells to enact antitumoural effects.

Interrogation of transcriptome data from NASH-related and non-NASH-related HCC patients identified the late neutrotime signature to be significantly enriched in NASH-HCC when compared with HCCs of other aetiologies^44^ (figure 6D). Moreover, the late neutrotime profile was specifically associated with human HCCs that are stratified by gene expression to the immune class and specifically within this group to the exhausted immune class which are typically resistant to immunotherapy (figure 6E).^44–47^ This suggests that TANs in human NASH-HCC resemble the mature phenotype of TANs in mouse NASH-HCC and may play a role in preventing ICI responses in patients, and as such we speculate they may be susceptible to similar therapeutic neutrotime reprogramming with AZD5069/anti-PD1 treatment. To examine the stage at which neutrophils are reprogrammed we compared intratumoural to circulating neutrophil profiles in the treatment groups in the DEN/ALIOS model. Neutrotime reprogramming was specific to the intratumoural population of AZD5069/anti-PD1 treated mice and noteably without an associated neutrotime change in circulating neutrophils of this treatment group (figure 6F), this observation being consistent with tumour-selective neutrophil reprogramming. Hence, while the combination therapy brings about reprogramming of TAN maturity, it leaves intact the mature phenotype of circulating neutrophils required for their classic anti-microbial surveillance functions.^48 49^

RNA-Seq of purified Ly6G^+^ neutrophils revealed that TANs from AZD5069/anti-PD1 treatment mice were enriched for process networks associated with cell cycle, phagocytosis and antigen presentation when compared with vehicle controls (figure 6G and online supplemental figure 7C). AZD5069 monotherapy modestly enhanced the expression of signatures associated with cell division, phagocytosis, and degranulation while also eliciting a reduction in protumour gene expression, with all of these effects being accentuated when AZD5069 was combined with anti-PD1 (figure 6G and online supplemental figure 7C–E). Anti-PD1 treatment promoted antigen presentation and processing signatures, which were also enriched in combined AZD5069/anti-PD1 treatment but not with AZD5069 monotherapy (figure 6G). These findings were again indicative of the combinatorial effects of AZD5069/anti-PD1 therapy on TAN phenotype. AZD5069 monotherapy (but not anti-PD1 monotherapy) suppressed the expression of key immune checkpoint molecules in TANs, including downregulation of *Cd80, Pvr, Sirpa, Pdl1* and *Pdl2*. This loss of immune checkpoint gene expression was maintained in the context of combination therapy and for some genes (eg, *Pvr* and *Srpa*) we noted more pronounced suppressive effects when compared with the AZD5069 montherapy alone (online supplemental figure 7F). Hence, TAN-enriched immune hubs observed in AZD5069/anti-PD1 treated tumours are able to avoid ICI signals that might otherwise cause immune exhaustion. AZD5069/anti-PD1 TANs also displayed a strong correlation with transcriptional changes seen in neutrophils during an acute systemic inflammatory response,^50^ including expression of genes involved in exocytosis, myeloid cell activation and degranulation (online supplemental figure 7G, H). Finally, these AZD5069/anti-PD1 TANs closely resembled a recently objectively characterised acute-inflammatory immature-Ly6G^Int^ neutrophil population isolated from lipopolysaccharide-(LPS)-treated mice^50^ (online supplemental figure 7I). In summary, AZD5069/anti-PD1 combination therapy brings about reprogramming of HCC-NASH TANs to exhibit immature, proliferative and inflammatory characteristics.

From these data we hypothesised that activated early neutrotime TANs have anti-tumoural properties. Due to their relatively low numbers and lack of specific surface markers it was not possible to isolate reprogrammed TANs from tumours in order to formally test this hypothesis. Instead, as proof-of-principle, we isolated inflammatory immature neutrophils enriched in the BM of LPS-treated mice and a pool of mature BM neutrophils isolated from control PBS treated mice. Adoptively transferring these cells to orthotopic NASH-HCC mice, we asked whether they would bring about an anti-tumoural effect in combination with anti-PD1 treatment (figure 6H and online supplemental figure 7J, K). Transfer of inflammatory immature neutrophils lead to a significant increase in circulating immature CXCR2^Lo^ neutrophils in the blood and resulted in a significant reduction in tumour burden (figure 6I and online supplemental figure 7L). In contrast transfer of mature neutrophils had no effect on tumour burden (figure 6I). To investigate underlying mechanism we examined intratumoural cDC1 and CD8^+^ T cells. Similar to treatment of mice with AZD5069/anti-PD1, we noted transfused immature neutrophils caused increased activation (CD86^+^) of intratumoural XCR1^+^ cDC1 cells and elevated CD8^+^ T cells in tumours, unlike mice transfused with equal numbers of mature neutrophils (figure 6J–L). Moreover, the adoptive transfer of neutrophils from LPS treated was associated with increased intratumoural Gzmb expression indicative of stimulation of cytotoxic activity within the tumour (figure 6M). Hence, we conclude that BM derived immature inflammatory neutrophils which have phenotypic similarities to AZD5069/anti-PD1 reprogrammed TANs are able to stimulate immune remodelling within HCC tumours and promote anti-tumoural effects.

## Discussion

Immune-based therapies hold considerable promise for the treatment of advanced HCC, however at present response rates are low and according to recent reports this is at least in-part determined by the immune cell composition of the tumour.^45 51 52^ HCC on the background of NASH presents additional considerations because of the crosstalk occurring between inflammatory cells and various metabolic adaptions manifest in the disease such as insulin resistance, steatosis, oxidative stress and altered mitochondrial function.^53^ Pfister and colleagues have reported that immunotherapy in NASH-HCC may be compromised due to high numbers of protumour CD8^+^PD1^+^ T cells in the tumour microenvironment.^9^ Here we show that selective targeting of neutrophils with a CXCR2 antagonist promotes the anti-tumour effects of anti-PD1 therapy in NASH-HCC, this effect being mechanistically associated with activation of classic CD8^+^ T cell and DC mediated anti-tumour immunity, but also with intratumoural reprogramming of TAN maturation and phenotype. Based on IMC we propose that the reprogrammed TANs, which are characterised by their proliferative and inflammatory characteristics, associate in tight clusters with CD8^+^ T cells and APCs to form anti-tumour Gzmb-secreting immune hubs within the NASH-HCC tumour microenvironment. Our work therefore emphasises the strong potential for targeted therapeutic manipulation of the innate immune system in cancer, but also uncovers a previously unrecognised crosstalk between the C-X-C chemokine/CXCR2 and PD1/PDL1 signalling systems that may be exploited to improve immunotherapy responses not only in NASH-HCC but also in other types of cancer that exhibit immunotherapy resistance.^54 55^

Neutrophil infiltration is a key pathological feature of human NASH that may result from upregulation of hepatic CXCL8 (IL-8) and CXCL1,^56 57^ which we also report here to be enriched in human NASH-HCC. In addition, expression of CXCR2 on neutrophils in NASH is selectively enhanced through an auto-stimulation mechanism involving the upregulation of neutrophil-derived lipocalin 2.^58^ Once present in the NASH and NASH-HCC microenvironments neutrophils are exposed to high levels of TGF-β which, as reported with other cancers,^19–21^ can polarise TANs towards a so-called ‘N2’ tumour-promoting state.^14^ It is also pertinent to address the relationship between TANs and myeloid-derived suppressor cells (MDSC), the latter being a heterogeneous population comprising polymorphonuclear granulocytic Ly6G^+^Ly6C^Lo^ (PMN-MDSC) and monocytic Ly6G^-^Ly6C^Hi^ (M-MDSC) cells. Accumulating evidence suggests that PMN-MDSC are immunosuppressive neutrophils and may be functionally very similar to the TANs that have been termed ‘N2’, with shared protumour properties.^14 59^ In the mouse there are no markers to distinguish between PMN-MDSCs and neutrophils and as such we cannot rule out that TANs in mouse models of NASH-HCC include PMN-MDSCs which may also be susceptible to reprogramming in response to combined CXCR2 antagonism and anti-PD1 therapy. However, the typical inhibitory effects on DC and CD8^+^ T cell functions associated with the activities of PMN-MDSCs and immunosuppressive neutrophils were clearly overcome by combined AZD5069/anti-PD1 therapy.

A growing body of evidence suggests that CXCR2 inhibition may be therapeutically beneficial in many human cancers including; pancreatic, lung, ovarian, prostate, colon and now the liver.^26–30 60^ Furthermore, in genetic murine models of lung cancer, inhibition of CXCR1 and 2 receptors in combination with anti-PD1 amplified anti-tumour responses.^61 62^ The proposed mechanism of action, until now, however, was thought to rely on reprogramming of the tumour immune microenvironment, primarily as a result of impaired myeloid recruitment. The most remarkable immunobiological finding of our study was that, paradoxically, when combined with anti-PD1, CXCR2 inhibition leads to an increase in tumour neutrophils and a selective reprogramming of the TAN neutrotime, with no evidence for a similar systemic effect on circulating neutrophils. The immature proliferative phenotype of the reprogrammed TANs evokes extramedullary granulopoiesis which can be seen in mice following antibody-mediated depletion of Ly6G^+^ cells and that is due to survival and expansion of residual tissue neutrophils driven by high systemic levels of granulocyte colony-stimulating factor,^63^ indeed this rebound effect meant that we were unable to exploit this protocol to directly interrogate the function of reprogrammed TANs. However, as proof-of-principle we were able to establish that adoptive transfer of immature activated neutrophils isolated from BM of LPS-treated mice has antitumour activity in NASH-HCC and this effect was accompanied by remodelling of tumour immunity including the activation of cDC1 cells, elevated CD8^+^ T cell counts and induction of anti-tumoural Gzmb; these being changes that were also noted with AZD506/anti-PD1 therapy. In future work it will be important to identify selective markers of the reprogrammed TANs that might be exploited for detailed functional characterisation, as well as for enabling their selective experimental manipulation which at present is not possible. Also, it will be important to determine precisely how and why combined CXCR2 antagonism and anti-PD1 treatment selectively induces proliferative immature neutrophils in the tumour. Clinically the ability to selectively reprogramme TANs while retaining mature anti-microbial neutrophils in the circulation may be very relevant in HCC since bacterial infections and septic shock are common clinical challenges in cirrhotic patients (in whom 90% of HCC develops).^64^

In summary, we present a novel combination immunotherapy that enhances the efficacy of anti-PD1 in NASH-HCC. As the CXCR2 antagonist AZD5069 has been demonstrated to be safe for use in humans it is timely to determine if HCC patients would benefit from a similar therapy.

## Methods

### Mice ethical approval

All animal experiments using the orthotopic NASH-HCC model and DEN/ALIOS model were performed in accordance with a UK Home Office licence (PP8854860, PP390857 and PP0604995), adhered to ARRIVE guidelines (https://www.nc3rs.org.uk/arrive-guidelines), and in accordance with the UK Animal (Scientific Procedures) Act 1986, and were subject to review by the animal welfare and ethical review board of the University of Glasgow and Newcastle University. All mice were housed in specific pathogen free conditions with unrestricted access to food and water and maintained on a constant 12 hours light-dark cycle under controlled climate (19°C–22°C, 45%–65% humidity). All animal experiments using the CD-HFD were performed in accordance with German law and the governmental bodies, and with approval from the Regierungspräsidium Karlsruhe (G11/16, G129/16, G7/17). Male mice were housed at the German Cancer Research Centre (DKFZ) (constant temperature of 20°C–24°C and 45%–65% humidity with a 12 hours light-dark cycle and were maintained under specific pathogen-free conditions.

### Quantification and statistical analysis

Statistical analyses were performed using GraphPad Prism software (V.9 GraphPad Software, La Jolla, CA, USA) and R (V.3.5.1) performing tests as indicated and were considered statistically significant. P values are included in figures.

Additional methods are described in online supplemental materials.

10.1136/gutjnl-2021-326259.supp2 Supplementary data

10.1136/gutjnl-2021-326259.supp3 Supplementary data

## Data Availability

Data are available in a public, open access repository. All data will be deposited with accession codes, unique identifiers or web links for publicly available datasets provided before publication.

## References

[1] Sung H , Ferlay J , Siegel RL , et al . Global cancer statistics 2020: GLOBOCAN estimates of incidence and mortality worldwide for 36 cancers in 185 countries. CA Cancer J Clin 2021;71:209–49. 10.3322/caac.21660 33538338

[2] Younossi Z , Stepanova M , Ong JP , et al . Nonalcoholic steatohepatitis is the fastest growing cause of hepatocellular carcinoma in liver transplant candidates. Clin Gastroenterol Hepatol 2019;17:748–55. 10.1016/j.cgh.2018.05.057 29908364

[3] Dyson J , Jaques B , Chattopadyhay D , et al . Hepatocellular cancer: the impact of obesity, type 2 diabetes and a multidisciplinary team. J Hepatol 2014;60:110–7. 10.1016/j.jhep.2013.08.011 23978719

[4] Gallage S , García-Beccaria M , Szydlowska M , et al . The therapeutic landscape of hepatocellular carcinoma. Med 2021;2:505–52. 10.1016/j.medj.2021.03.002 35590232

[5] Finn RS , Qin S , Ikeda M , et al . Atezolizumab plus bevacizumab in unresectable hepatocellular carcinoma. N Engl J Med 2020;382:1894–905. 10.1056/NEJMoa1915745 32402160

[6] Yau T , Kang Y-K , Kim T-Y , et al . Efficacy and safety of nivolumab plus ipilimumab in patients with advanced hepatocellular carcinoma previously treated with sorafenib: the CheckMate 040 randomized clinical trial. JAMA Oncol 2020;6:e204564. 10.1001/jamaoncol.2020.4564 33001135 PMC7530824

[7] Finn RS , Ryoo B-Y , Merle P , et al . Pembrolizumab as second-line therapy in patients with advanced hepatocellular carcinoma in KEYNOTE-240: a randomized, double-blind, phase III trial. J Clin Oncol 2020;38:193–202. 10.1200/JCO.19.01307 31790344

[8] Yau T , Park J-W , Finn RS , et al . Nivolumab versus sorafenib in advanced hepatocellular carcinoma (CheckMate 459): a randomised, multicentre, open-label, phase 3 trial. Lancet Oncol 2022;23:77–90. 10.1016/S1470-2045(21)00604-5 34914889

[9] Pfister D , Núñez NG , Pinyol R , et al . NASH limits anti-tumour surveillance in immunotherapy-treated HCC. Nature 2021;592:450–6. 10.1038/s41586-021-03362-0 33762733 PMC8046670

[10] Haber PK , Puigvehí M , Castet F , et al . Evidence-based management of hepatocellular carcinoma: systematic review and meta-analysis of randomized controlled trials (2002-2020). Gastroenterology 2021;161:879–98. 10.1053/j.gastro.2021.06.008 34126063 PMC12276942

[11] Sheng J , Zhang J , Wang L , et al . Topological analysis of hepatocellular carcinoma tumour microenvironment based on imaging mass cytometry reveals cellular neighbourhood regulated reversely by macrophages with different ontogeny. Gut 2022;71:1176–91. 10.1136/gutjnl-2021-324339 34253573

[12] Shaul ME , Fridlender ZG . Tumour-associated neutrophils in patients with cancer. Nat Rev Clin Oncol 2019;16:601–20. 10.1038/s41571-019-0222-4 31160735

[13] Coffelt SB , Wellenstein MD , de Visser KE . Neutrophils in cancer: neutral no more. Nat Rev Cancer 2016;16:431–46. 10.1038/nrc.2016.52 27282249

[14] Geh D , Leslie J , Rumney R . Neutrophils as potential therapeutic targets in hepatocellular carcinoma. Nat Rev Gastroenterol Hepatol 2022:1–17. 35022608 10.1038/s41575-021-00568-5

[15] Tetri LH , Basaranoglu M , Brunt EM , et al . Severe NAFLD with hepatic necroinflammatory changes in mice fed trans fats and a high-fructose corn syrup equivalent. Am J Physiol Gastrointest Liver Physiol 2008;295:987–95. 10.1152/ajpgi.90272.2008 PMC4059366 18772365

[16] Zaki MYW , Mahdi AK , Patman GL , et al . Key features of the environment promoting liver cancer in the absence of cirrhosis. Sci Rep 2021;11:16727. 10.1038/s41598-021-96076-2 34408183 PMC8373870

[17] Margetts J , Ogle LF , Chan SL , et al . Neutrophils: driving progression and poor prognosis in hepatocellular carcinoma? Br J Cancer 2018;118:248–57. 10.1038/bjc.2017.386 29123264 PMC5785742

[18] Esteban-Fabró R , Willoughby CE , Piqué-Gili M , et al . Cabozantinib enhances the efficacy and immune modulatory activity of anti-PD1 therapy in a syngeneic mouse model of hepatocellular carcinoma. J Hepatol 2020;73:S40. 10.1016/S0168-8278(20)30632-2

[19] Shaul ME , Levy L , Sun J , et al . Tumor-associated neutrophils display a distinct N1 profile following TGFβ modulation: a transcriptomics analysis of pro- vs. antitumor TANs. Oncoimmunology 2016;5:e1232221. 10.1080/2162402X.2016.1232221 27999744 PMC5139653

[20] Fridlender ZG , Albelda SM . Tumor-associated neutrophils: friend or foe? Carcinogenesis 2012;33:949–55. 10.1093/carcin/bgs123 22425643

[21] Fridlender ZG , Sun J , Kim S , et al . Polarization of tumor-associated neutrophil phenotype by TGF-beta: "N1" versus "N2" TAN. Cancer Cell 2009;16:183–94. 10.1016/j.ccr.2009.06.017 19732719 PMC2754404

[22] Govaere O , Cockell S , Tiniakos D , et al . Transcriptomic profiling across the nonalcoholic fatty liver disease spectrum reveals gene signatures for steatohepatitis and fibrosis. Sci Transl Med 2020;12:aba4448. 10.1126/scitranslmed.aba4448 33268509

[23] Pinyol R , Torrecilla S , Wang H , et al . Molecular characterisation of hepatocellular carcinoma in patients with non-alcoholic steatohepatitis. J Hepatol 2021;75:865–78. 10.1016/j.jhep.2021.04.049 33992698 PMC12164395

[24] Jamieson T , Clarke M , Steele CW , et al . Inhibition of CXCR2 profoundly suppresses inflammation-driven and spontaneous tumorigenesis. J Clin Invest 2012;122:3127–44. 10.1172/JCI61067 22922255 PMC3428079

[25] Ha SY , Choi M , Lee T , et al . The prognostic role of mitotic index in hepatocellular carcinoma patients after curative hepatectomy. Cancer Res Treat 2016;48:180–9. 10.4143/crt.2014.321 25797572 PMC4720078

[26] Yang G , Rosen DG , Liu G , et al . CXCR2 promotes ovarian cancer growth through dysregulated cell cycle, diminished apoptosis, and enhanced angiogenesis. Clin Cancer Res 2010;16:3875–86. 10.1158/1078-0432.CCR-10-0483 20505188 PMC2930833

[27] Steele CW , Karim SA , Leach JDG , et al . CXCR2 inhibition profoundly suppresses metastases and augments immunotherapy in pancreatic ductal adenocarcinoma. Cancer Cell 2016;29:832–45. 10.1016/j.ccell.2016.04.014 27265504 PMC4912354

[28] Li Y , He Y , Butler W , et al . Targeting cellular heterogeneity with CXCR2 blockade for the treatment of therapy-resistant prostate cancer. Sci Transl Med 2019;11:aax0428. 10.1126/scitranslmed.aax0428 PMC7238624 31801883

[29] Cheng Y , Mo F , Li Q , et al . Targeting CXCR2 inhibits the progression of lung cancer and promotes therapeutic effect of cisplatin. Mol Cancer 2021;20:62. 10.1186/s12943-021-01355-1 33814009 PMC8019513

[30] Katoh H , Wang D , Daikoku T , et al . CXCR2-expressing myeloid-derived suppressor cells are essential to promote colitis-associated tumorigenesis. Cancer Cell 2013;24:631–44. 10.1016/j.ccr.2013.10.009 24229710 PMC3928012

[31] Tang KH , Li S , Khodadadi-Jamayran A , et al . Combined inhibition of SHP2 and CXCR1/2 promotes antitumor T-cell response in NSCLC. Cancer Discov 2022;12:47–61. 10.1158/2159-8290.CD-21-0369 34353854 PMC8758507

[32] Hurkmans DP , Basak EA , Schepers N , et al . Granzyme B is correlated with clinical outcome after PD-1 blockade in patients with stage IV non-small-cell lung cancer. J Immunother Cancer 2020;8. 10.1136/jitc-2020-000586 PMC7254154 32461348

[33] Rousalova I , Krepela E . Granzyme B-induced apoptosis in cancer cells and its regulation (review). Int J Oncol 2010;37:1361–78. 10.3892/ijo_00000788 21042704

[34] Wagner C , Iking-Konert C , Denefleh B , et al . Granzyme B and perforin: constitutive expression in human polymorphonuclear neutrophils. Blood 2004;103:1099–104. 10.1182/blood-2003-04-1069 14512315

[35] Nowacki TM , Kuerten S , Zhang W , et al . Granzyme B production distinguishes recently activated CD8(+) memory cells from resting memory cells. Cell Immunol 2007;247:36–48. 10.1016/j.cellimm.2007.07.004 17825804 PMC2134935

[36] Gardner A , de Mingo Pulido Álvaro , Ruffell B . Dendritic cells and their role in immunotherapy. Front Immunol 2020;11:924. 10.3389/fimmu.2020.00924 32508825 PMC7253577

[37] Dyer DP , Medina-Ruiz L , Bartolini R , et al . Chemokine receptor redundancy and specificity are context dependent. Immunity 2019;50:378–89. 10.1016/j.immuni.2019.01.009 30784579 PMC6382461

[38] Deczkowska A , David E , Ramadori P , et al . XCR1^+^ type 1 conventional dendritic cells drive liver pathology in non-alcoholic steatohepatitis. Nat Med 2021;27:1043–54. 10.1038/s41591-021-01344-3 34017133

[39] Zhu YP , Padgett L , Dinh HQ , et al . Identification of an early Unipotent neutrophil progenitor with pro-tumoral activity in mouse and human bone marrow. Cell Rep 2018;24:2329–41. 10.1016/j.celrep.2018.07.097 30157427 PMC6542273

[40] Grieshaber-Bouyer R , Radtke FA , Cunin P , et al . The neutrotime transcriptional signature defines a single continuum of neutrophils across biological compartments. Nat Commun 2021;12:2856. 10.1038/s41467-021-22973-9 34001893 PMC8129206

[41] Actor JK , Hwang S-A , Kruzel ML . Lactoferrin as a natural immune modulator. Curr Pharm Des 2009;15:1956–73. 10.2174/138161209788453202 19519436 PMC2915836

[42] Spadaro M , Montone M , Arigoni M , et al . Recombinant human lactoferrin induces human and mouse dendritic cell maturation via Toll-like receptors 2 and 4. Faseb J 2014;28:416–29. 10.1096/fj.13-229591 24088817

[43] Cutone A , Rosa L , Ianiro G , et al . Lactoferrin’s anti-cancer properties: safety, selectivity, and wide range of action. Biomolecules 2020;10:10030456. 10.3390/biom10030456 PMC7175311 32183434

[44] Villanueva A , Portela A , Sayols S , et al . DNA methylation-based prognosis and epidrivers in hepatocellular carcinoma. Hepatology 2015;61:1945–56. 10.1002/hep.27732 25645722 PMC12337117

[45] Sia D , Jiao Y , Martinez-Quetglas I , et al . Identification of an immune-specific class of hepatocellular carcinoma, based on molecular features. Gastroenterology 2017;153:812–26. 10.1053/j.gastro.2017.06.007 28624577 PMC12166766

[46] Ayers M , Lunceford J , Nebozhyn M , et al . IFN-γ-related mRNA profile predicts clinical response to PD-1 blockade. J Clin Invest 2017;127:2930–40. 10.1172/JCI91190 28650338 PMC5531419

[47] Sangro B , Melero I , Wadhawan S , et al . Association of inflammatory biomarkers with clinical outcomes in nivolumab-treated patients with advanced hepatocellular carcinoma. J Hepatol 2020;73:1460–9. 10.1016/j.jhep.2020.07.026 32710922 PMC7751218

[48] Mayadas TN , Cullere X , Lowell CA . The multifaceted functions of neutrophils. Annu Rev Pathol 2014;9:181–218. 10.1146/annurev-pathol-020712-164023 24050624 PMC4277181

[49] Amulic B , Cazalet C , Hayes GL , et al . Neutrophil function: from mechanisms to disease. Annu Rev Immunol 2012;30:459–89. 10.1146/annurev-immunol-020711-074942 22224774

[50] Mackey JBG , McFarlane AJ , Jamieson T . Maturation, developmental site, and pathology dictate murine neutrophil function. bioRxiv.

[51] Kurebayashi Y , Ojima H , Tsujikawa H , et al . Landscape of immune microenvironment in hepatocellular carcinoma and its additional impact on histological and molecular classification. Hepatology 2018;68:1025–41. 10.1002/hep.29904 29603348

[52] Yarchoan M , Xing D , Luan L , et al . Characterization of the immune microenvironment in hepatocellular carcinoma. Clin Cancer Res 2017;23:7333–9. 10.1158/1078-0432.CCR-17-0950 28928158 PMC5881396

[53] Anstee QM , Reeves HL , Kotsiliti E , et al . From NASH to HCC: current concepts and future challenges. Nat Rev Gastroenterol Hepatol 2019;16:411–28. 10.1038/s41575-019-0145-7 31028350

[54] Boi SK , Orlandella RM , Gibson JT , et al . Obesity diminishes response to PD-1-based immunotherapies in renal cancer. J Immunother Cancer 2020;8:e000725. 10.1136/jitc-2020-000725 33427691 PMC7757487

[55] An Y , Wu Z , Wang N , et al . Association between body mass index and survival outcomes for cancer patients treated with immune checkpoint inhibitors: a systematic review and meta-analysis. J Transl Med 2020;18:235. 10.1186/s12967-020-02404-x 32532255 PMC7291531

[56] Pan X , Chiwanda Kaminga A , Liu A , et al . Chemokines in non-alcoholic fatty liver disease: a systematic review and network meta-analysis. Front Immunol 2020;11:01802. 10.3389/fimmu.2020.01802 PMC7530185 33042108

[57] Liu K , Wang F-S , Xu R . Neutrophils in liver diseases: pathogenesis and therapeutic targets. Cell Mol Immunol 2021;18:38–44. 10.1038/s41423-020-00560-0 33159158 PMC7852892

[58] Ye D , Yang K , Zang S , et al . Lipocalin-2 mediates non-alcoholic steatohepatitis by promoting neutrophil-macrophage crosstalk via the induction of CXCR2. J Hepatol 2016;65:988–97. 10.1016/j.jhep.2016.05.041 27266617

[59] Zhou J , Nefedova Y , Lei A , et al . Neutrophils and PMN-MDSC: their biological role and interaction with stromal cells. Semin Immunol 2018;35:19–28. 10.1016/j.smim.2017.12.004 29254756 PMC5866202

[60] Tang KH , Li S , Khodadadi-Jamayran A . Combined inhibition of SHP2 and CXCR1/2 promotes anti-tumor T cell response in NSCLC. Cancer Discov 2021. 10.1158/2159-8290.CD-21-0369 PMC8758507 34353854

[61] Kargl J , Zhu X , Zhang H , et al . Neutrophil content predicts lymphocyte depletion and anti-PD1 treatment failure in NSCLC. JCI Insight 2019;4:130850. 10.1172/jci.insight.130850 31852845 PMC6975266

[62] Sun L , Clavijo PE , Robbins Y , et al . Inhibiting myeloid-derived suppressor cell trafficking enhances T cell immunotherapy. JCI Insight 2019;4:126853. 10.1172/jci.insight.126853 30944253 PMC6483637

[63] Moses K , Klein JC , Männ L , et al . Survival of residual neutrophils and accelerated myelopoiesis limit the efficacy of antibody-mediated depletion of Ly-6G+ cells in tumor-bearing mice. J Leukoc Biol 2016;99:811–23. 10.1189/jlb.1HI0715-289R 26819319

[64] Bajaj JS , Kamath PS , Reddy KR . The evolving challenge of infections in cirrhosis. N Engl J Med 2021;384:2317–30. 10.1056/NEJMra2021808 34133861

