# Supplementary material for: CXCR2 inhibition enables NASH-HCC immunotherapy
Source: Gut. 2022 Apr 27;71(10):2093–106. doi: 10.1136/gutjnl-2021-326259 (PMC9484388; doi:10.1136/gutjnl-2021-326259)
Supplement: Supplementary data [file gutjnl-2021-326259supp001.pdf]

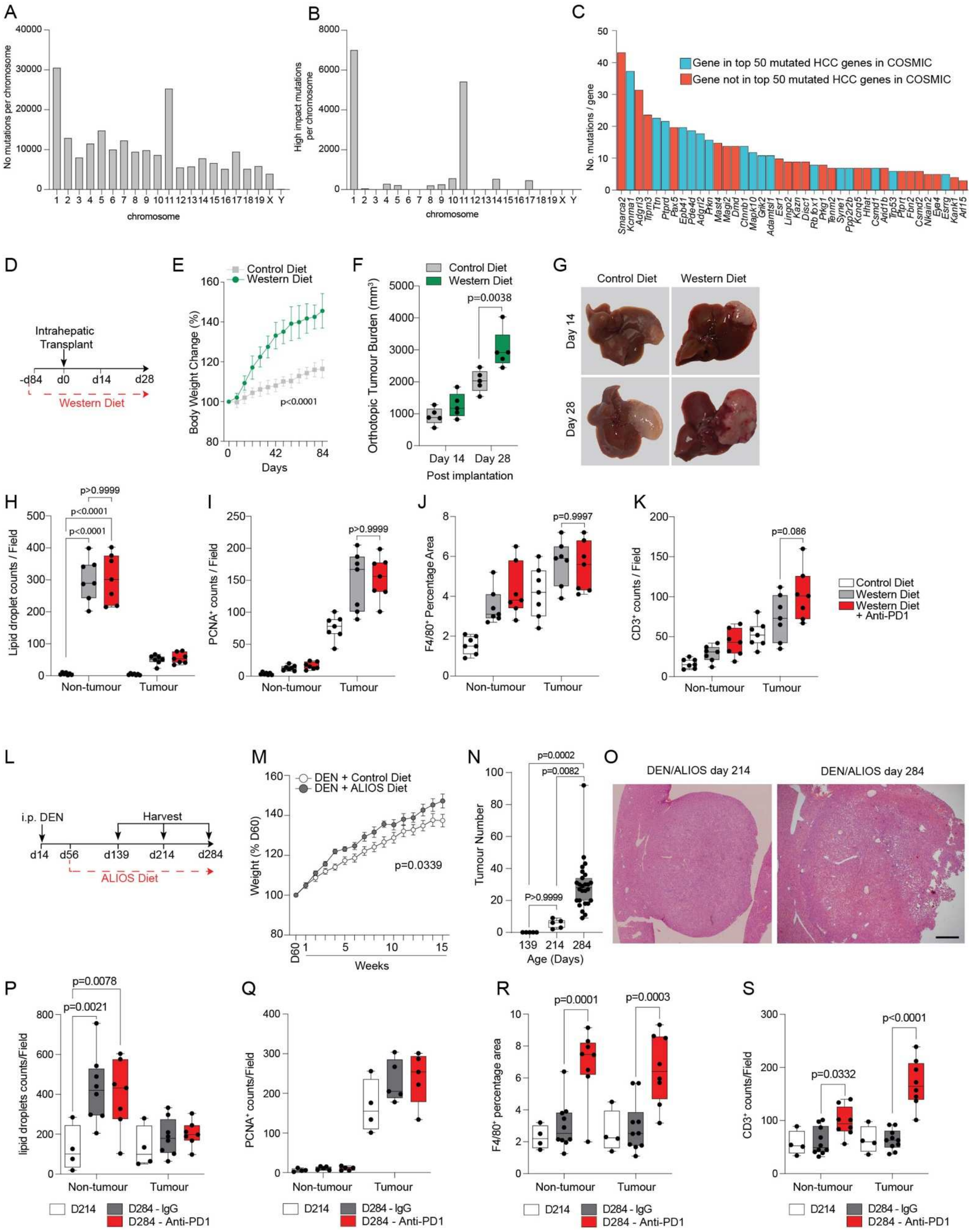

Supplementary Figure 1. Steatosis promotes tumour development and anti-PD1 resistance.
A) Number of mutations per chromosome in Hep53.4 HCC cells. **B)** Number of high impact mutations per chromosome in Hep53.4 cells. **C)** Top 40 mutated genes in Hep53.4 HCC cells, with high confidence human orthologues. Blue: gene in top 50 mutated HCC genes in the Catalogue of Somatic Mutations in Cancer (COSMIC) database. Red: gene not in top 50 mutated HCC genes in COSMIC. **D)** Timeline schematic of the orthotopic NASH-HCC model. **E)** Quantification of body weight change for mice fed a normal or Western diet, presented as percentage weight change compared with pre-diet weight. Mean ± SEM. **F)** Quantification of the tumour burden for the orthotopic NASH-HCC mice fed a control or Western diet at day 14 and day 28 post intra-hepatic injection. **G)** Representative images of livers at day 14 and day 28 post-intrahepatic injection for the orthotopic NASH-HCC mice fed a control or Western diet. **H-K)** Quantification of lipid droplet, PCNA⁺, F4/80⁺ and CD3⁺ counts/field in non-tumour liver and tumour of the orthotopic NASH-HCC mice fed a control or Western diet and treated with IgG-control or anti-PD1, at day 28 post intrahepatic injection. **L)** Timeline schematic of the autochthonous DEN/ALIOS NASH-HCC model. **M)** Quantification of body weight change for mice injected with DEN at day 14 and fed a control or ALIOS diet from day 60, presented as a percentage of weight change compared with day 60. **N)** Quantification of tumour number for DEN/ALIOS mice at day 139, 214 and 284. **O)** [illegible]

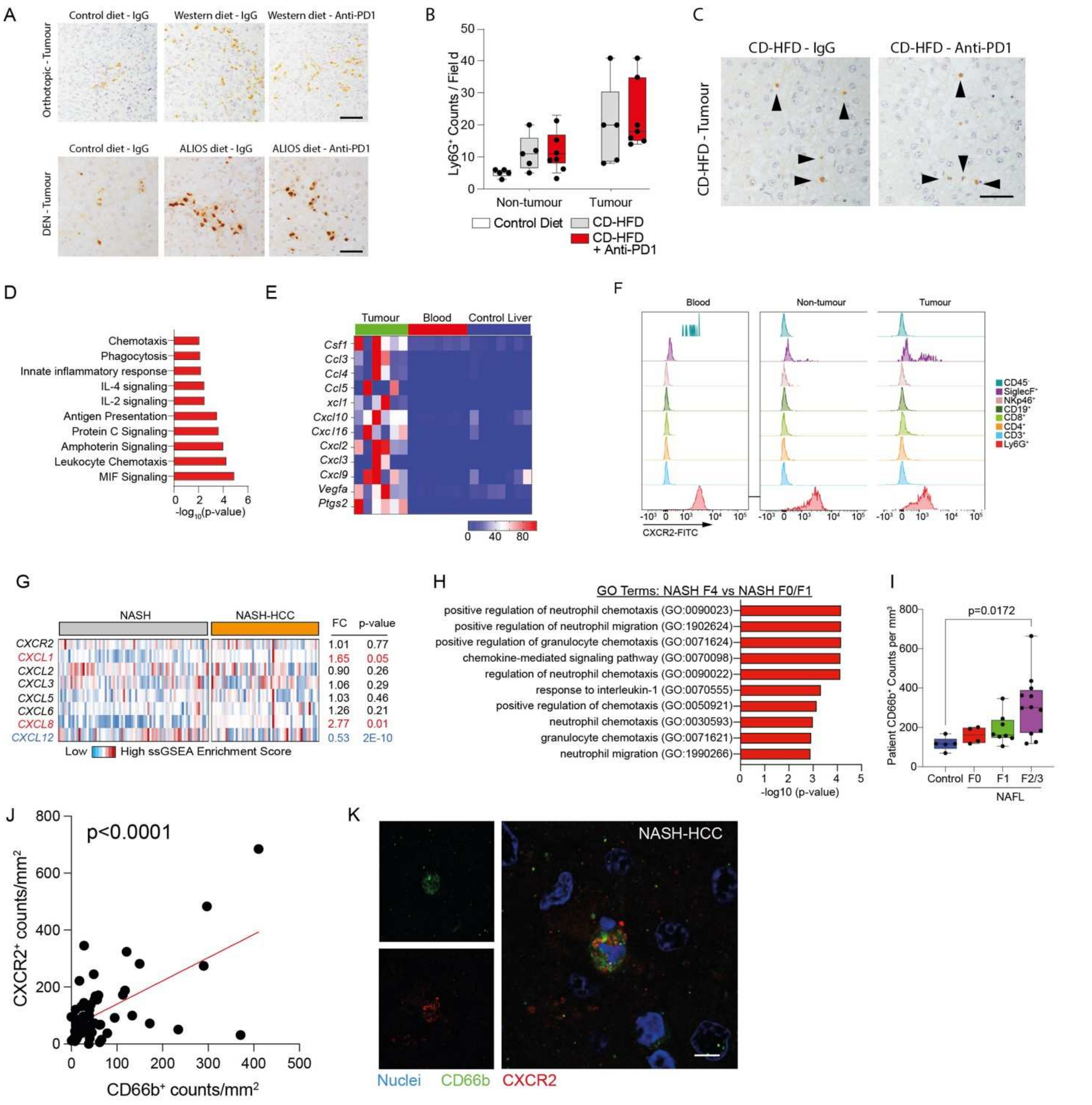

Supplementary Figure 2. Neutrophils associate with NASH-HCC in mouse models and humans

A) Representative image of Ly6G$^+$ neutrophil staining in tumours from the orthotopic NASH-HCC mice fed a control or western diet (top) and DEN mice fed a control or ALIOS diet (bottom). Scale bar = 100 µm. **B, C)** Quantification and representative images (tumour only) of Ly6G$^+$ counts/field in non-tumour liver and tumours from IgG-control or anti-PD1 treated choline-deficient high fat diet (CD-HFD) mice. **D)** Top 10 process networks enriched in DEGs with increased expression in DEN/ALIOS TANs compared with peripheral blood and control liver Ly6G$^+$ neutrophils. **E)** Heatmap showing row-scaled expression of pro-tumour associated neutrophil genes in DEN/ALIOS TANs compared with peripheral blood and control liver neutrophils. **F)** Representative histograms for CXCR2 staining in immune cell populations isolated from the peripheral blood, non-tumour liver and tumours of DEN/ALIOS mice. **G)** Heatmap showing row-scaled expression of *Cxcl* chemokine transcripts, related to neutrophil-function, for human NASH compared with NASH-HCC. NASH n=74 individuals; NASH-HCC n=53 individuals. **H)** GO Terms enriched in advanced (F4) compared with early stage (F0/F1) NASH patients. Data are from patient resected tissue RNA-Seq. Data accessed

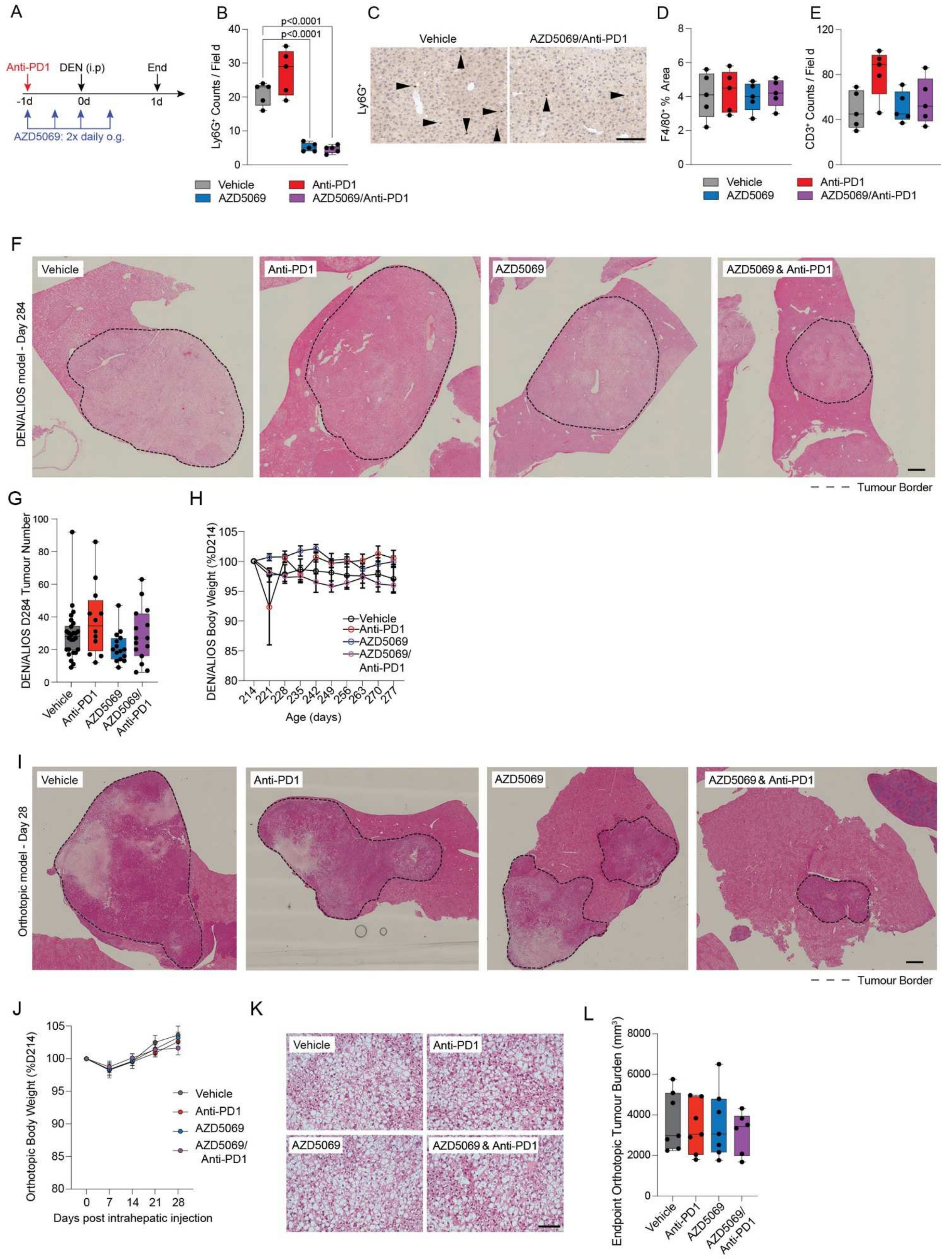

Supplementary Figure 3. CXCR2 inhibition alters neutrophil regulation and sensitises to anti-PD1 therapy
A) Schematic for the acute-DEN model treatment regime. **B)** Quantification Ly6G⁺ counts/field by IHC for the livers from acute-DEN mice treated with Vehicle-control, anti-PD1, AZD5069, or AZD5069/anti-PD1. **C)** Representative Ly6G⁺ staining of liver sections from acute-DEN mice. Scale bar = 100 µm. **D)** Quantification of F4/80⁺ macrophages as a percentage area of the field by IHC for the livers from acute-DEN mice treated with Vehicle-control, anti-PD1, AZD5069, or AZD5069/anti-PD1. **E)** Quantification of CD3⁺ counts/field by IHC for the livers from acute-DEN mice treated with Vehicle-control, anti-PD1, AZD5069, or AZD5069/anti-PD1. **F)** Representative image of H&E stained liver sections for DEN/ALIOS mice at day 284. Scale bar = 1,000 µm. **G)** Quantification of tumour number for DEN/ALIOS mice at day 284 for each treatment arm. **H)** Quantification of body weight change for DEN/ALIOS mice, presented as percentage of weight change compared with pre-treatment start (day 214). Mean ± SEM. **I)** Representative image of H&E stained liver sections from the orthotopic NASH-HCC mice at day 28. Scale bar = 1,000 µm. **J)** Quantification of body weight change for the orthotopic NASH-HCC mice presented as a percentage of weight change compared with

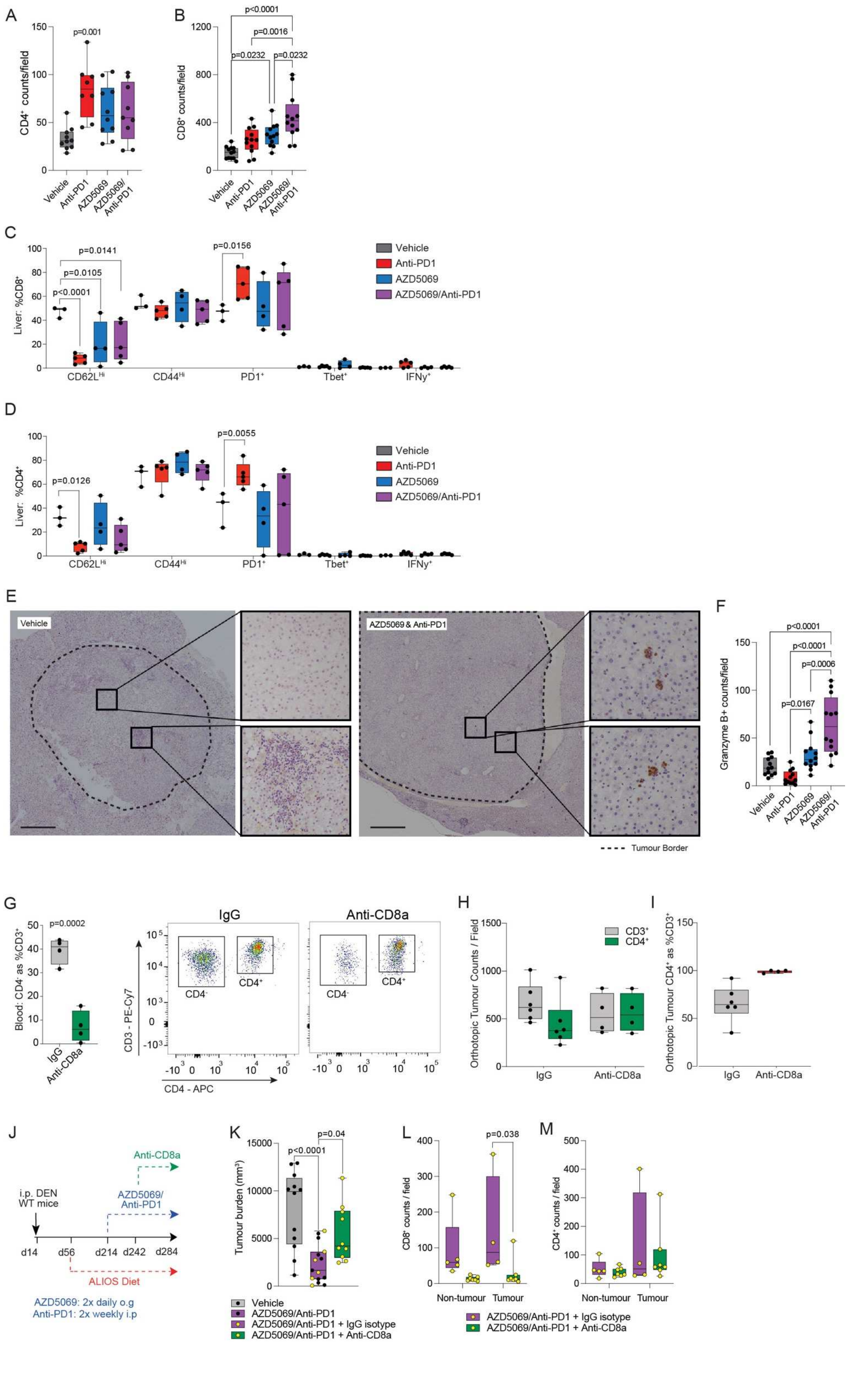

Supplementary Figure 4. Cytotoxic CD8$^+$ T cells contribute to combined AZD5069 & anti-PD1 anti-tumour effect

A, B) Quantification of CD4$^+$ and CD8$^+$ counts/field in tumours DEN/ALIOS model for each treatment arm. **C, D)** Quantification of CD3$^+$CD8$^+$ and CD3$^+$CD4$^+$ cell surface phenotyping for DEN/ALIOS mice from each treatment arm at day 284. **E)** Representative images of granzyme B$^+$ clusters in Vehicle-control and AZD5069/anti-PD1 treated DEN/ALIOS livers. Scale bar = 1,000 µm. **F)** Quantification of Granzyme B$^+$ counts/field in livers for the orthotopic NASH-HCC mice for each treatment arm. **G)** Quantification and representative flow cytometry plots of CD4$^-$ as a percentage of CD3$^+$ cells in the peripheral blood for the orthotopic NASH-HCC mice treated with AZD5069/anti-PD1 and IgG-control or anti-CD8α. **H)** Quantification of CD3$^+$ and CD4$^+$ cells by IHC analysis in the tumours for the orthotopic NASH-HCC mice treated with AZD5069/anti-PD1 and IgG-control or anti-CD8α. **I)** Quantification of CD4$^+$ as a percentage of CD3$^+$ cells in tumours for the orthotopic NASH-HCC mice treated with AZD5069/anti-PD1 and IgG-control or anti-CD8α. **J)** Timeline schematic for the anti-CD8a depletion regime in the autochthonous DEN/ALIOS model. **K)** Quantification of tumour burden for DEN/ALIOS mice at day 284 for each treatment arm (Vehicle data from Figure 3b). **L, M)** Quantification of CD8$^+$ and CD4$^+$ counts/field in non-tumour and tumour for DEN/ALIOS mice from each treatment arm. Dots in **(A-D, F-I, K-M)** represent individual mice. Significance tested using: One Way ANOVA

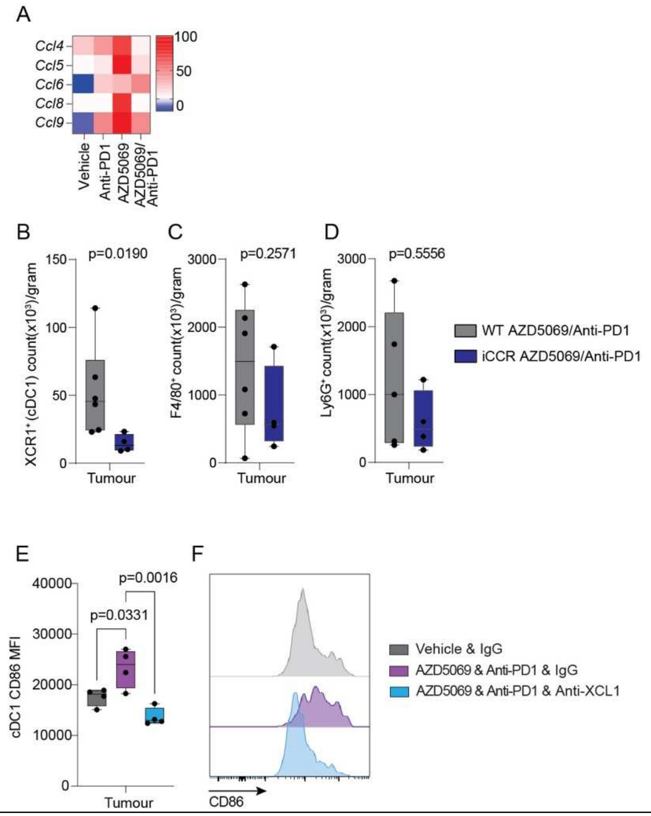
A
Ccl4
Ccl5
Ccl6
Ccl8
Ccl9
100
50
0
Vehicle
Anti-PD1
AZD5069
AZD5069/ Anti-PD1
B
p=0.0190
XCR1+ (cDC1) count(x10³)/gram
Tumour
C
p=0.2571
F4/80+ count(x10³)/gram
Tumour
D
p=0.5556
Ly6G+ count(x10³)/gram
Tumour
WT AZD5069/Anti-PD1
iCCR AZD5069/Anti-PD1
E
p=0.0016
p=0.0331
cDC1 CD86 MFI
Tumour
F
CD86
Vehicle & IgG
AZD5069 & Anti-PD1 & IgG
AZD5069 & Anti-PD1 & Anti-XCL1

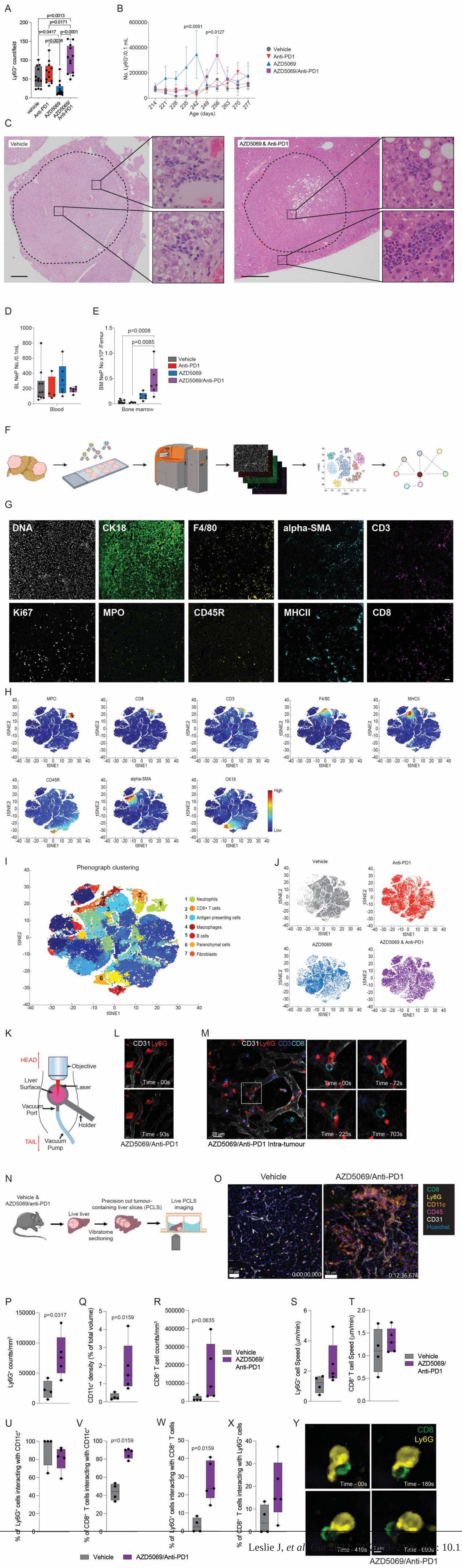

Supplementary Figure 6. Imaging mass cytometry and live cell imaging reveals intra-tumour proliferating

A) Quantification of Ly6G⁺ counts/field in livers for the orthotopic NASH-HCC mice for each treatment arm. **B)** Flow cytometric quantification of the number of Ly6G⁺/0.1 mL peripheral blood from serial tail bleed analysis for DEN/ALIOS mice between day 214 and day 277. Mean ± SEM. **C)** Representative images of H&E staining in livers for DEN/ALIOS Vehicle and AZD5069/anti-PD1 treated mice. Clusters of neutrophils with a banded morphology are enlarged in AZD5069/anti-PD1 treated mice. Scale bar = 1,000 µm. **D, E)** Flow cytometric quantification of NeP count in 0.1ml blood and bone marrow (BM) for DEN/ALIOS mice for each treatment arm at day 284. **F)** Schematic of Fluidigm Hyperion imaging mass cytometry pipeline, including preparation of tumour tissue microarray, metal conjugated antibody staining, imaging mass cytometry and downstream analysis including tSNE and neighbourhood analysis performed using histoCAT. **G)** Representative images of hyperion staining for DNA intercalator, CK18, F4/80, alpha-SMA, CD3, Ki67, MPO, CD45R, MHCII and CD8. Scale bar = 100 µm. **H)** tSNE plots showing clustering of MPO, CD8, CD3, F4/80, MHCII, CD45R, alpha-SMA and CK18 positive cells. **I)** tSNE plot showing phenograph clustering of cell populations across all images and treatment groups. **J)** tSNE plots showing the contribution of individual treatment groups to clustering. **K)** Schematic for DEN/ALIOS model liver intravital microscopy (IVM) set-up. **L)** Representative images for intra-tumour extravascular Ly6G⁺ clusters by IVM for DEN/ALIOS mice treated with AZD5069/anti-PD1 at day 284. **M)** Representative images for intra-tumour IVM for DEN/ALIOS mice treated with AZD5069/anti-PD1 at day 284 (right). Data are representative of n=1 mouse. Scale bar = 20 µm. **N)** Schematic for DEN/ALIOS model precision cut tumour-containing liver slice (PCLS) microscopy set-up. **O)** Representative images of live cell imaging of CD8, Ly6G, CD11c, CD45, CD31 and Hoechst in PCLS generated from DEN/ALIOS mice treated with either vehicle or AZD5069/anti-PD1 at day 284. Scale bar = 50 µm. **P-R)** Quantification of Ly6G⁺ counts/mm^3, CD11c⁺ density as a percentage of total volume, CD8⁺ T cell counts/mm^3 in PCLS generated from DEN/ALIOS mice treated with either vehicle or AZD5069/anti-PD1 at day 284. **S, T)** Quantification of Ly6G⁺ and CD8⁺ T cell speed (µm/min) in PCLS generated from DEN/ALIOS mice treated with either vehicle or AZD5069/anti-PD1 at day 284. **U)** Quantification of the percentage of Ly6G⁺ cells interacting with CD11c⁺ cell surface in PCLS generated from DEN/ALIOS mice treated with either vehicle or AZD5069/anti-PD1 at day 284. **V)** Quantification of the percentage of CD8⁺ T cells interacting with CD11c⁺ cell surface in PCLS generated from DEN/ALIOS mice treated with either vehicle or AZD5069/anti-PD1 at day 284. **W)**

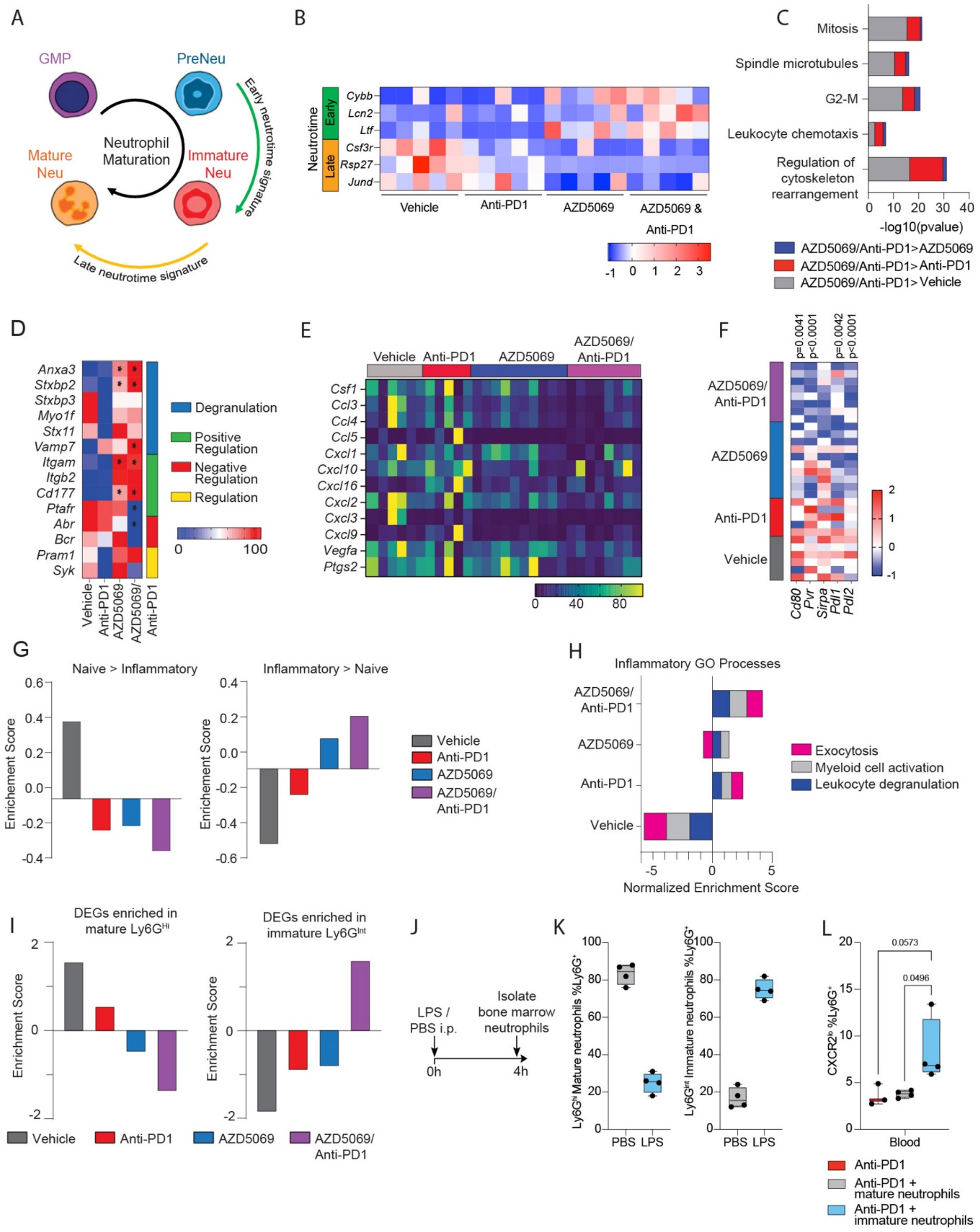

Supplementary Figure 7. Combined AZD5069 and anti-PD1 therapy promotes an immature inflammatory tumour-associated neutrophil.

A) Schematic depicting neutrophil maturation stages classified according to early and late neutrotime signatures (as described by Grieshaber-Bouyer *et al.*). **B)** Heatmap showing normalised expression of selected early and late neutrotime genes in neutrophils isolated from treated orthotopic NASH-HCC mice at day 28. **C)** GSEA for process networks enriched in DEN/ALIOS TANs from AZD5069/anti-PD1 treated compared with Vehicle, anti-PD1 and AZD5069. **D)** Heatmap showing row-scaled expression of genes associated with neutrophil degranulation GO Processes (0043312, 0043313, 0043314, 0043315) for DEN/ALIOS mice TANs. * denotes p<0.05 significance. **E)** Heatmap showing row-scaled expression of genes associated with a pro-tumour neutrophil phenotype for DEN/ALIOS TANs from each treatment arm. **F)** Heatmap showing row-scaled expression of immune checkpoint genes for DEN/ALIOS TANs. **G)** GSEA from DEGs increased in neutrophils isolated from PBS-control (left) and LPS-treated mice (right) for TANs isolated from DEN/ALIOS mice for each treatment arm. **H)** GSEA for inflammatory GO Processes enriched in LPS-treated compared with PBS-control mice for TANs isolated from DEN/ALIOS mice for each treatment arm. **I)** GSEA for DEGs upregulated in LPS-treated peripheral blood, compared with PBS-controls, by mature Ly6G^{Hi} neutrophils (left) and immature Ly6G^{int} neutrophils (right) for DEN/ALIOS TANs. DEGs identified from bulk hepatic Ly6G^{+} neutrophils from PBS-control and LPS-treated mice analysed by RNA-Seq. **J)** Schematic showing timeline of treatment of WT mice with LPS or PBS for harvesting of immature and mature neutrophils. **K)** Flow cytometric quantification of Ly6G^{hi} and Ly6G^{int} neutrophils as a percentage of total Ly6G^{+} bone marrow cells from LPS and PBS treated mice. **L)** Flow cytometric quantification of CXCR2^{Lo} neutrophils as a percentage of Ly6G^{+} neutrophils from the
