# Supplementary material for: CXCR2 inhibition enables NASH-HCC immunotherapy
Source: Gut. 2022 Apr 27;71(10):2093–106. doi: 10.1136/gutjnl-2021-326259 (PMC9484388; doi:10.1136/gutjnl-2021-326259)
Supplement: Supplementary data [file gutjnl-2021-326259supp002.pdf]

Supplementary methods

Mice diets and treatments

For the orthotopic NASH-HCC model, intrahepatic injection of Hep-53.4 cells into the left lobe of C57BL/6 mice was performed under isoflurane general anaesthesia. Mice were fed *ad libitum* either a normal chow diet plus drinking water or a modified western diet (Envigo -TD.120528) plus sugar water (23.1 g/L fructose and 18.9 g/L glucose) for 3 months prior to implantation. For clinical relevance CXCR2smi and anti-PD1 therapeutic intervention was started at 14 days post implantation when small macroscopic tumours are present. Mice were then harvested at 28 days post implantation or left to reach an approved humane endpoint. To deplete $CD8^+$ cells, mice received either anti-CD8a (Biolegend, 53-6.7) or IgG control (Biolegend, RTK2758; twice weekly i.p. 200μg) for 7 days after the initial 7 days of treatment with CXCR2smi and anti-PD1. Neutralization of XCL1 to deplete cDC1 cells; 50 μg of anti-XCL1 (R&D systems MAB486) or isotype-matched control antibodies (R&D systems MAB006) were injected i.p. twice a week for 2 weeks in combination with AZD5069/anti-PD1 therapy. Immature bone marrow neutrophil enrichment was performed with a single dose of lipopolysaccharide (LPS) from *E. coli*_0111:B4; (1 mg/kg, i.p.) or PBS control and femurs collected after 4 hours. Polymorphonuclear fraction of bone marrow from PBS and LPS treated mice isolated by percol density centrifugation. High density fraction, containing predominantly neutrophils was washed, resuspended, counted and $1x10^7$ cells i.v. injected twice weekly for 2 weeks into orthtopic NASH-HCC anti-PD1 treated mice. Tumour burden was calculated by measuring the size of the tumour in three perpendicular planes using digital callipers.

For the DEN/ALIOS model, WT C57BL/6 mice, bred in house were injected with a single dose of DEN at 80 mg/kg by i.p. injection at 14 days of age. Mice were placed on either the ALIOS diet consisting of an irradiated high trans-fat diet composed of 22% hydrogenated vegetable (Envigo, TD.110201) and sugar water or a control diet at 60 days of age. For clinical relevance, treatment with AZD5069 (AstraZeneca; 250 mg/mL in 0.5% Hydroxypropyl Methylcellulose (HPMC), 0.1% Tween 80; 100 mg/kg, o.g.) twice daily, or vehicle (0.5% HPMC, 0.1% Tween 80; o.g.) twice daily, anti-PD1 (Biolegend, RMP1-14; 200 μg, i.p.) bi-weekly or IgG (Biolegend, RTK2758; 200 μg, i.p.) bi-weekly was used to treat the mice. To deplete $CD8^+$ cells in AZD5069/anti-PD1 DEN/ALIOS mice, mice received either anti-CD8a (Biolegend, 53-6.7) or IgG control (Biolegend, RTK2758; first dose i.p. 400μg and then twice weekly i.p. 200μg) from day 242 until harvest at day 284.

For the long-time CD-HFD feeding model, 5-week-old C57BL/6 mice (male) were fed choline-deficient high-fat diet (CD-HFD) (Research Diets; D05010402) for 13 months to induce NASH-HCC. For

therapeutic intervention, anti-PD1 (BioXcell, RMP1-14; 200 µg, i.p.) bi-weekly, IgG (BioXcell, LTF-2; 200 µg, i.p.) bi-weekly, or vehicle (PBS; i.p.) bi-weekly, was used to treat the mice for 8 weeks.

For acute-DEN experiments, 12-15 week old WT C57B6/J mice bred in house or ordered in from Charles River were treated with a single dose of anti-PD1 or IgG control (dosing as above for DEN/ALIOS model) and/or AZD5069 or vehicle control (dosing as above for DEN/ALIOS model) twice daily. The following day mice received a single high-dose DEN injection (100 mg/kg, i.p.) and tissues were harvested the following morning.

For PBS-control and LPS-induced acute-inflammatory models, data is described in full in Mackey, *et al.,* 2021. Briefly, 8-10 week old WT C57BL/6 mice ordered in from Charles River were injected with a single dose of PBS (i.p) or LPS (*E. coli,* 0111:B4; 1 mg/kg, i.p.) and tissues were harvested after 24 hours.

Scoring of tumour burden

For the DEN/ALIOS model, whole livers were weighed, then dissected into 3-4 sections and liver tumours were scored using digital callipers. For the orthotopic NASH-HCC model, whole livers were weighed and tumours were scored with digital callipers in three dimensions for calculating tumour volume (mm^3).

Sample processing and staining for flow cytometry and FACS

Tissues were collected in ice-cold PBS. Blood samples were collected into EDTA coated syringes and immediately treated with Erythrocyte Lysis Buffer containing NH_4Cl, $KHCO_3$ and Na_2EDTA (Sigma-Aldrich) in dH_2O, pH 7.2-7.4. Non-tumour liver and tumours were manually diced. Tumours were digested using a mouse tumour dissociation kit in GentleMACS C digestion tubes with a GentleMACS tissue dissociator (Miltenyi Biotec). Enzyme activity was neutralised by addition of cold RPMI/2% FBS and suspension was dispersed through a 70 µm cell strainer. Single cell suspensions were treated with RBC lysis buffer. Cells were blocked with CD16/32 (BioLegend) as required and stained with directly conjugated antibodies (listed below) for 25 minutes at 4 °C in the dark in PBS/1% BSA/0.05% NaN_3. Zombie NIR (zNIR) fixable viability (1:1000; BioLegend) was added to exclude dead cells. For surface antigen staining only, cells were fixed in 2% paraformaldehyde. For intracellular staining, cells were fixed and permeabilized using the FOXP3/Transcription Factor staining buffer set (ThermoFisher), then staining of intracellular proteins. For cell counts, 10,000 AccuCount fluorescent particles (Spherotech) were added to each sample. All experiments were performed using a BD LSRFortessa flow cytometer using BD FACSDive^{TM} Diva software. Data were analysed using FlowJo software version 10.7.1.

All antibodies were purchased from BioLegend, except for CD101-PE, CD101-PE/Cy7, CD45-SB600 and IFNγ-PE/Cy7, which were obtained from eBioscience and Siglec-F-APC/Cy7, Siglec-F-Bv605, Ly6G-Buv395, CD11b-FITC, and CD162-Bv510, which were obtained from BD Biosciences.

General immune panel I: CXCR2-FITC (1:100, SA04E51), CD45-Bv711 (1:200, 30-F11), Siglec-F-Bv605 (1:200, E50-2440), CD31-PerCPCy5.5 (1:100, 390), CD3ε-Bv421 (1:100, 145-2C11), Ly6G-Buv395 (1:100, 1A8), CD8α-AF700 (1:100, 53-6.7), CD19-AF647 (1:100, 6D5), CD4-PE/Cy7 (1:200, RM4-5), Nkp46-PE (1:100, 29A1.4), fixable viability dye zNIR.

General immune panel II: CXCR2-FITC (1:100, SA04E51), XCR1-Bv785 (1:100, ZET), Ly6C-Bv711 (1:200, HK1.4), CD11b-Bv650 (1:400, M1/70), CD11c-Bv605 (1:200, N418), F4/80-Bv510 (1:200, BM8), CD115-Bv421 (1:200, AFS98), CD45-AF700 (1:200, 30-F11), CD172a-AF647 (1:100, P84), CD64-PE/Cy7 (1:200, X54-5/7.1), IA/IE-Bv421 (1:200, M5/114.15.2), CD26-PE (1:200, H194-112), fixable viability dye zNIR.

Tail bleed panel: Siglec-F-APC/Cy7 (1:200, E50-2440), CD45-Bv711 (1:200, 30-F11), CD62L-Buv395 (1:200, MEL-14), CD101-PE/Cy7 (1:200, Moushi101), Ly6G-AF647 (1:400, 1A8), CD11b-Bv650 (1:400, M1/70), CXCR2-FITC (1:100, SA04E51), CD3ε-Bv421 (1:100, 145-2C11), CD4-Bv605 (1:100, RM4-5), CD8α-AF700 (1:100, 53-6.7), PD-1-Bv785 (1:100, 29F.1A12), CD44-PerCP/Cy5.5 (1:100, IM7), PD-L1-PE (1:100, 10F.9G2), fixable viability dye zNIR.

Neutrophil panel: Siglec-F-APC/Cy7 (1:200, E50-2440), CD117-PE/Cy7 (1:100, 2B8), Ly6G-AF647 (1:400, 1A8), CD11b-Bv650 (1:400, M1/70), CXCR2-FITC (1:100, SA04E51), CD101 (1:200, Moushi101), CD45-Bv711 (1:200, 30-F11), PD-1-Bv785 (1:100, 29F.1A12), CD62L-Buv395 (1:200, MEL-14), fixable viability dye zNIR.

T cell panel: CD44-PerCP/Cy5.5 (1:100, IM7), CD4-FITC (1:100, RM4-5), PD-1-Bv785 (1:100, 29F.1A12), T-bet-Bv711 (1:200, 4B10), IL-17-Bv650 (1:400, TC11-18H10.1), CD45-SB600 (1:200, 30-F11), CD3ε-Bv421 (1:100, 145-2C11), CD19-Buv805 (1:400, 6D5), CD62L-Buv395 (1:200, MEL-14), CD8α-AF700 (1:100, 53-6.7), GranzymeB-AF647 (1:50, GB11), IFNγ-PE/Cy7 (1:200, XMG1.2), CD69-PE (1:100, H1.2F3) fixable viability dye zNIR.

DC and macrophage panel: CD45-AF700 (1:200, 30-F11), CD11c-Bv605 (1:200, N418), CD26-PE (1:200, H194-112), XCR1-Bv785 (1:100, ZET), CD103-PerCP/Cy5.5 (1:100, 2e7), IA/IE-Bv421 (1:200, M5/114.15.2), CD86-FITC (1:200, GL-1), CD11b-Bv650 (1:400, M1/70), CD172a-AF647 (1:100, P84),

Ly6C-Bv711 (1:200, HK1.4), F4/80-Bv510 (1:200, BM8), CD64-PE/Cy7 (1:200, X54-5/7.1), fixable viability dye zNIR.

NeP panel: Siglec-F-Bv605 (1:200, E50-2440), FcεR1α-AF647 (1:400, MAR-1), CD16/32-PerCP/Cy5.5 (1:100, 93), Ly6B-FITC (1:400, 74), CD11a-PE (1:400, M17/4), Ly6G-PE/Cy7 (1:400, 1A8), CD162-Bv510 (1:400, 2PH1), CD115-Bv421 (1:200, AFS98), fixable viability dye zNIR.

Fluorescent-Activated Cell Sorting (FACS) panel: CD45-SB600 (1:200, 30-F11), CD48-PE/Cy7 (1:200, HM48-1), Ly6G-AF647 (1:400, 1A8), CD11b-FITC (1:400, M1/70), CD3ε-PE (1:100, 145-2C11), DAPI.

Neutrophil and T cell RNA isolation and sequencing and analysis

Ly6G$^+$ neutrophils and CD3$^+$ T cells were FACS-sorted from the peripheral blood and tumours of DEN/ALIOS mice. Purity of isolated populations was analysed by flow cytometry at ≥ 97%. RNA was isolated using the Rneasy Micro Kit (Qiagen) according to the manufacturer's protocol. RNA quality and quantity was checked on an Agilent Bioanalyser 2100 using RNA Pico 6000 chip. Libraries for cluster generation and DNA sequencing were prepared following the TaKaRa SMARTer Stranded Total RNA-Seq Kit- Pico Input Mammalian v2 protocol. Quality and quantity of the DNA libraries was assessed on an Agilent 2200 Tapestation (D1000 screentape) and Qubit (Thermo Fisher Scientific) respectively. The libraries were run on the Illumina Next Seq 500 using the High Output v2.5, 75 cycles kit (2 x 36cycles, paired-end reads). Illumina data were demultiplexed using bcl2fastq version 2.19.0, then adaptor sequences were removed using Cutadapt version 0.6.4 and quality checked using fastqc version 0.11.8. Next, paired end reads were aligned to the mouse genome version GRCm38.95 using HISAT2 version 2.1.0, and gene expression was determined using Htseq version 0.11.2. Differential expression analysis was performed using the R package DESeq2 version 1.22.2. Accurately identified genes were classed as those with ≥ 2 reads/million in ≥ half of the biological replicas in at least one experimental condition. DEGs were identified as those with a p-value ≤ 0.05 and fold change ≥ 1.5 between compared data sets. Gene ontology and pathway analysis was performed using MetaCore (Clarivate Analytics).

Laser capture micro-dissection RNA-Sequencing

Formalin-fixed, paraffin-embedded (FFPE) 10μm liver sections on Zeiss membrane slides were dewaxed, rehydrated through graded alcohols, and then stained with haematoxylin and eosin (H&E). Tumour tissue was excised using Zeiss PALM microbeam laser capture microdissection microscope. RNA was then isolated using the High Pure FFPE RNA Micro Kit (Roche). RNA quality and quantity was

checked using the DV200 metric (REF) on an Agilent Bioanalyser 2100 using RNA Pico 6000 chip. Total RNA sequencing libraries were prepared using the SMART-Seq Stranded kit [Takara Bio] following the manufacturer's protocol. Libraries were quantified using and a Tapestation 4200 [Agilent] and Qubit 4 [Life Technologies] and equimolar pooled and sequenced at >30 million 100 bp single reads per sample on a NovaSeq 6000 using an 100 cycle SP flow cell [Illumina]. Data for individual samples was demultiplexed into separate FASTQ files using Illumina's bcl2fastq software. Data were analysed as above (Neutrophil RNA isolation and sequencing and analysis).

Whole tumour RNA-Sequencing and Analysis

Whole tumor and healthy tissue was snap frozen and stored at -80C. Tissue was homogenized using the Precellys Evolution homogenizer and bulk RNA was isolated using the Rneasy Kit (Qiagen) according to the manufacturer's instructions, including the optional Dnase I step. RNA quality and quantity was analysed on a Nanodrop 2000 (Thermo Fisher Scientific) and an Agilent 2200 TapeStation (D1000 screentape). Only samples with a RIN value >7 were used for library preparation. Libraries were prepared using the TruSeq stranded mRNA Kit. Library quality and quantity were assessed using 2200 TapeStation (Agilent) and Qubit (ThermoFisher Scientific). The libraries were then run on an Illumina NextSeq 500 using the High Output 75 cycles kit (2x36cycle paired end reads). Data were analysed as above (Neutrophil RNA isolation and sequencing and analysis).

RNA isolation, cDNA synthesis and qRT-PCR

Ly6G^{+} neutrophils sorted from orthtopic NASH-HCC tumours were snap frozen and stored at -80°c. RNA was isolated using the Rneasy Kit (Qiagen) according to the manufacturer's instructions, including the optional Dnase I step. cDNA synthesis was performed using the iScript cDNA synthesis kit (Bio-Rad) according to the manufacturer's instructions. Real time PCR was performed using SYBR Green jumpstart ready mix and the primers listed in (**Supplementary Table 1**).

Publicly available gene expression dataset analysis

Publicly available RNA-seq datasets of differentially expressed genes from RNA-seq performed on biopsies from patients with NASH F0/F1 and NASH F4 were accessed[1]. Gene ontology analysis was performed using genes significantly upregulated in patients with advanced disease. NASH-related HCCs, non-NASH-HCCs, as well as NASH liver human samples were previously described[2,3]. Transcriptomic data from human NASH liver (n=74) and NASH-HCC (n=53) samples were used to assess the expression of CXCR2 and key neutrophil-related cytokines in both tissues. The single sample Gene

Set Enrichment Analysis (ssGSEA) module of GenePattern was used to determine the enrichment scores of immune-related gene signatures and signatures associated to response to immune checkpoint inhibitors[2,3]. Differentially expressed genes from RNA-seq performed on peripheral blood and liver Ly6G^{+} neutrophils, and peripheral blood Ly6G^{Int} and Ly6G^{Hi} neutrophils, from LPS-treated acute systemic inflammatory response and PBS-control treated mice were accessed. Gene ontology and pathway analysis was performed using MetaCore (Clarivate Analytics).

Hep53.4 DNA isolation and analysis

DNA was isolated from Hep53.4 cells using the QIAamp DNA kit (Qiagen) as per manufacturers instruction and then sent for exome sequencing (Novogene). Raw read outputs were then passed through FastQC and FastQ screen for quality control. Raw reads were then aligned to the mm10 genome assembly using the Burrow-Wheeler aligner (BWA-MEM) software. Duplicate reads were identified using Picard tools and base recalibration was performed using BaseRecalibrator. Variants were called using Mutect2, before analysis using Ensembl Variant Effect Predictor to identify the likely effects of genomic variants. Data were visualised using R version 4.0.2 using the maftools, tidyverse packages and MutationalPatterns packages. We extracted known drivers of HCC in human disease from the Catalogue of Somatic Mutations in Cancer (COSMIC) database. Using mouse:human homology mapping through ENSEMBL cross species annotations, we then identified mutations present in known cancer driver genes. To account for gene length, the number of mutations is presented as number of mutations per million base pairs.

Histology and immunohistochemistry

FFPE tissue sections were stained with H&E using established protocols. Immunohistochemistry (IHC) was performed on FFPE sections that were dewaxed and rehydrated through graded alcohols. Endogenous peroxidases were then blocked using hydrogen peroxide/methanol solution. Antigen retrieval was performed using Tris-EDTA pH9. Endogenous avidin and biotin were blocked using the Avidin/Biotin blocking kit (Vector Laboratories, SP-2001) and non-specific binding was blocked using 20% swine serum in PBS. Antibodies were incubated at room temperature for 1 hour. Sections were washed and incubated in the appropriate biotinylated secondary antibody. Sections were then washed and incubated in Vectastain Elite ABC-HRP reagent (Vector Laboratories, PK-6100). Staining was visualised using DAB substrate kite (Vector Laboratories, SK-4100), counter stained with mayer haematoxylin and then mounted. CD3 (AbCam Ab16669, pH6 1:50), CD4 (eBioscience 14-9766-82, ER2 Leica, 1:500), CD8 (eBioscience 14-0808-82, ER2 Leica, 1:500), Ly6G (clone 1A8, 2B Scientific BE0075-1, ER2 Leica, 1:60000), LTF (Thermofisher, PA5-95513, 1:200), granzyme B (Abcam, ab255598, Clone:

EPR22645-206, 1:100), CD8 (Abcam, ab217344, Clone: EPR21769, 1:100). Image analysis was performed using a Nikon Eclipse Upright microscope and NIS-Elements BR analysis software. A minimum of ten consecutive non-overlapping fields were imaged where possible.

Automated IHC and analysis of human tissue

Automated immunohistochemistry was performed using the Ventana Discovery XT platform. FFPE sections were dewaxed and rehydrated in EZ prep solution and then Tris-EDTA heat mediated antigen retrieval was performed. Endogenous peroxides and proteins were blocked using the Discovery Inhibitor CM. Sections were then incubated with primary antibodies (CD66b – Biolegend 305102, CXCR2 – Sigma HPA031999) followed by the appropriate secondaries. Staining was visualised by incubated slides in DAB followed by counterstaining with haematoxylin and then mounting. Sections were scanned using a Leica Aperio scanner and the analysis performed using Aperio ImageScope slide viewing software. Cell counts were performed on the whole tissue samples and normalised to the total area analysed (mm^2).

Imaging Mass Cytometry

The following antibodies were used for imaging mass cytometry: CK18 (Thermofisher, PA5-14263), F4/80 (Biorad, Cl:A3-1), CD3 (Biorad, CD3-12), Ki67 (Thermofisher, 14-5698-82), CD8 (Abcam, EPR21769), CD45R (Thermofisher, 14-0452-82), MHCII (Thermofisher, 14-5321-81), αSMA (ProteinTech, 14395-1-AP) and MPO (R&D, AF3667). Ly6G was not used to identify neutrophils as it was not compatible with IMC, instead MPO was used. Antibodies were first validated by immunohistochemistry and immunofluorescence staining of FFPE tissue samples using a single antigen retrieval method consisting of heat mediated antigen retrieval using the universal antigen retrieval solution (Abcam). Based on the relative immunofluorescence signal antibodies were ranked and then pair with the appropriate metal for conjugation to maximise signal to noise ratio. Antibodies were conjugated to metals using MaxPar antibody conjugation kits (Fluidigm) following manufacturer's instructions. An antibody stabilisation solution was added before storage at 4°C (Candor Bioscience). The following metal-antibody conjugates were used: Nd145-CK18, Gd155-F4/80, Dy162-CD3, Dy163-Ki67. Dy164-CD8, Er166-CD45R, Tm169-MHCII, Er170-αSMA and Yb172-MPO. Conjugated antibodies were then validated signal by IMC in suspension mode (Helios) with antibody capture beads (AbC Total compensation beads, Thermo Fisher). To confirm conjugation did not alter antibody binding efficiency, antibodies were validated once more by immunofluorescence.

Tumour tissue microarrays were dewaxed and rehydrated through clearene and graded alcohols. Antigen retrieval was performed by immersing the slides in HIER Universal Antigen Retrieval reagent (Abcam, ab208572) at 95°C for 20 min. Slides were then washed and blocked with 3% BSA in 1X PBS for 45 min. The cocktail of antibodies was prepared at the proper concentration of each one by diluting it in 0.5% BSA in 1X PBS and leaving it overnight at 4°C in a humidified tray. The slides were then washed in 0.2% Triton X – 100 in PBS with gentle agitation, followed by rounds of 1X PBS. Nuclei were stained using DNA intercalator (Ir191/193) at 0.313μM in PBS for 30min. Finally, the slides were washed in ultra-pure water with gentle agitation and left air-drying at room temperature.

The Hyperion Tissue Imaging module was aligned and coupled to the Helios mass cytometry instrument and calibrated using the appropriate protocols (Fluidigm). Slide libraries were generated using low resolution images to aid identification of regions of interest (ROIs). ROIs ($1mm^2$ in area) were ablated from each tumour sample on the TMAs. Imaging data files were exported in MCD viewer software (Fluidigm) as 16-bit single layer TIFFs. Single cell segmentation was performed using the Bodenmiller pipeline, combining open source software Ilastik for machine learning-based pixel classification and Cell Profiler for actual single cell segmentation. Cell phenotype and interaction analysis was performed in HistoCat.

Liver intravital microscopy

Mice were anaesthetised and maintained using isoflurane in approximately 95% oxygen enriched air generated using a medical oxygen scavenger (VetTech). Mice were placed on a heat mat at 37 °C for the duration of the procedure. After loss of reflexes, the liver was exposed and a custom-built vacuum chamber fitted with a 13 mm glass cover slip placed onto the liver. Minimal suction (0.1-0.3 bar) was applied to stabilise the liver against the coverslip. Imaging was performed using an upright Zeiss LSM 880 Airyscan confocal microscope using a 20x/1 NA water immersion objective lens. Images were acquired in using a 32 channel Gallium arsenide phosphide spectral detector and signal was collected with a resolution of 8.9 nm over the visible spectrum. For visualisation of the vasculature and immune cell subtypes, fluorescently conjugated antibodies Ly6G (Biolegend, 5 μg, 1A8), CD45 (Biolegend, 5 μg, 30-F11), CD101 (eBioscience, 5 μg, Moushi101); CD3 (Biolegend, 5 μg, 145-2C11), CD8 (Biolegend, 5 μg, 53-6.7), CD31 (Biolegend, 10 μg, 390), were injected i.v. through the tail vein prior to anaesthesia. Livers were imaged for up to 60 minutes with a z-stack of 12 μm. At the end of the imaging session, mice were humanely killed by cervical dislocation under anaesthesia. Spectral images were unmixed with Zen software (Carl Zeiss) using references spectra acquired from unstained tissue (tissue autofluorescence) or slides labelled with individual fluorescently conjugated antibodies. Time-lapse

images were visualized and analysed using IMARIS software (Bitplane, Oxford Instruments, Abingdon UK; v9). For interaction analysis, spots were initially automatically created for $Ly6G^+$ cells and then corrected manually. Surfaces were initially automatically created for $CD3^+$ cells and then corrected manually. $Ly6G^+$ spots within 10µm, measured from the cell centre, of a $CD3^+$ surface were considered interacting.

Live precision cut tumour-containing liver slices (PCLS) imaging

Live PCLS procedure was adapted from McCowan et al for use with liver tissue[4]. Livers were sliced into 300µm thick sections on a vibratome and stained with Hoechst (1:2000) and directly conjugated fluorescent antibodies CD31 (Biolegend, 1:100, 390), CD8 (Biolegend, 1:100, 53-6.7), Ly6G (Novus bio, 1:100, 1A8), CD11c (Biolegend, 1:100, N418) and CD45 (Biolegend, 1:100, 30-F11) in complete medium (phenol-red free DMEM substituted with 1% FBS) for 20 minutes at 37°C. Slices were imaged on a Zeiss LSM880 confocal microscope in a full incubation chamber at 37°C with 5% CO2. Liver slices were imaged for 15-40min with z-stacks of 30µm. Acquisition was performed with a 32 channel Gallium arsenide phosphide(GaAsP) spectral detector using 20× objective. Samples were excited simultaneously with 405, 488, 561 and 633nm wavelength laser lines and signal was collected onto a linear array of the 32 GaAsp detectors in lambda mode with a resolution of 8.9 nm over the visible spectrum. Spectral images were then unmixed with Zen software (Carl Zeiss) using reference spectra acquired from unstained tissues (tissue autofluorescence) or beads labelled with single fluorophores.

4D images analysis

Timelapse images analysis and visualization was performed using Imaris (Bitplane). Neutrophils and T cells were detected and tracked using the 'spot detection' tool using either $Ly6G^+$ fluorescence intensities (neutrophils) or CD8 ($CD8^+$ T cells). $CD11c^+$ areas were segmented using the 'surface' tool to measure total $CD11c^+$ volume. All spots and surfaces were checked manually to avoid any false detections. Cell behaviour was determined using the track speed (indicating cell mobility). Interacting cells were determined as cells located < 15µm to other spots or surfaces.

Data Availability

All data will be deposited with accession codes, unique identifiers or web links for publicly available datasets provided before publication.

References

1. [dataset] Govaere O, Cockell S, Tiniakos D, Queen R, Younes R, Vacca M, et al. Transcriptomic profiling across the nonalcoholic fatty liver disease spectrum reveals gene signatures for steatohepatitis and fibrosis. *Sci Transl Med*. 2020;12(572). doi:10.1126/scitranslmed.aba4448. NCBI GEO repository (accession GSE135251), April 28, 2021
2. [dataset] Pinyol R, Torrecilla S, Wang H, Montironi C, Piqué-Gili M, Torres-Martin M, et al. Molecular characterisation of hepatocellular carcinoma in patients with non-alcoholic steatohepatitis. *J Hepatol*. 2021;75(4):865-878. doi:10.1016/j.jhep.2021.04.049. NCBI GEO repository (accession GSE63898, GSE15654 and GSE164760), April 14, 2020
3. [dataset] Villanueva A, Portela A, Sayols S, Battiston C, Hoshida Y, Méndez-González J, et al. DNA methylation-based prognosis and epidrivers in hepatocellular carcinoma. *Hepatology*. 2015;61(6):1945-1956. doi:10.1002/hep.27732. NCBI GEO repository (accession GSE56588), March 22, 2019
4. McCowan J, Fercoq F, Kirkwood PM, T'Jonck W, Hegarty LM, Mawer CM, et al. The transcription factor EGR2 is indispensable for tissue-specific imprinting of alveolar macrophages in health and tissue repair. *Sci Immunol*. 2021;6(65):eabj2132. doi:10.1126/sciimmunol.abj2132
