# Supplementary material for: CXCR2 inhibition enables NASH-HCC immunotherapy
Source: Gut. 2022 Apr 27;71(10):2093–106. doi: 10.1136/gutjnl-2021-326259 (PMC9484388; doi:10.1136/gutjnl-2021-326259)
Supplement: Supplementary data [file gutjnl-2021-326259supp003.pdf]

Table 1

	Forward primer	Reverse primer
Cybb	GATGATAGCACTGCACACCG	ATTCCTGTGATCCCAGCCAA
Lcn2	AAGGTGGCAGAACGAGATGA	ACCGCATAGTAGTGAGTCCG
Ltf	ACTGAATGGGTGGTGAGTGT	GGGAGTGCTGGCCAAATAAG
Csf3r	AAGACCCCAGGAGACCTTTG	GCCAGAGACAGAGACACACT
Rsp27	GACGTGAAATGCCCAGGATG	CTTTCAGTGCTGCTTCCTCC
Jund	CACGCTCTGCCTTTCCTTTT	AAAGAGAGGGGATGGTGTCG
